# Metabolomic and transcriptomice analyses of flavonoid biosynthesis in apricot fruits

**DOI:** 10.3389/fpls.2023.1210309

**Published:** 2023-07-18

**Authors:** Yilin Chen, Wenwen Li, Kai Jia, Kang Liao, Liqiang Liu, Guoquan Fan, Shikui Zhang, Yatong Wang

**Affiliations:** ^1^ College of Horticulture, Xinjiang Agricultural University, Urumqi, China; ^2^ Postdoctoral Research Station of Crop Science, Xinjiang Agricultural University, Urumqi, China; ^3^ Luntai Fruit Tree Resource Nursery, Xinjiang Academy of Agricultural Sciences, Luntai, China

**Keywords:** apricot, metabolomics, transcriptomics, flavonoid biosynthesis, weighted gene coexpression network analysis

## Abstract

**Introduction:**

Flavonoids, as secondary metabolites in plants, play important roles in many biological processes and responses to environmental factors.

**Methods:**

Apricot fruits are rich in flavonoid compounds, and in this study, we performed a combined metabolomic and transcriptomic analysis of orange flesh (JN) and white flesh (ZS) apricot fruits.

**Results and discussion:**

A total of 222 differentially accumulated flavonoids (DAFs) and 15855 differentially expressed genes (DEGs) involved in flavonoid biosynthesis were identified. The biosynthesis of flavonoids in apricot fruit may be regulated by 17 enzyme-encoding genes, namely PAL (2), 4CL (9), C4H (1), HCT (15), C3’H (4), CHS (2), CHI (3), F3H (1), F3’H (CYP75B1) (2), F3’5’H (4), DFR (4), LAR (1), FLS (3), ANS (9), ANR (2), UGT79B1 (6) and CYP81E (2). A structural gene-transcription factor (TF) correlation analysis yielded 3 TFs (2 bHLH, 1 MYB) highly correlated with 2 structural genes. In addition, we obtained 26 candidate genes involved in the biosynthesis of 8 differentially accumulated flavonoids metabolites in ZS by weighted gene coexpression network analysis. The candidate genes and transcription factors identified in this study will provide a highly valuable molecular basis for the in-depth study of flavonoid biosynthesis in apricot fruits.

## Introduction

As secondary metabolites of plants, flavonoids are widely found in natural polyphenols in vegetables, fruits, grains and tea, and play an important role in many biological processes and responses to environmental factors in plants (Shen et al., 2022). Flavonoids can affect the growth and development of plants and help plants resist biotic and abiotic stresses (Kai-Jie et al., 2022). For humans, flavonoids also exhibit antioxidant, free radical scavenging, anticancer, cholesterol-lowering and antibacterial functions (Gürler et al., 2020; Tao et al., 2022). Flavonoids with different chemical structures include flavonols, flavones, isoflavones, anthocyanins, flavanols, dihydroflavones and chalcones. Flavonols and isoflavones can regulate the transport of auxin and thus regulate the growth and development of plants. Flavonoids and isoflavones can also be used as chemical attractants of rhizobia in plant rhizobial symbionts or inducers of nitrogen-fixing nodules. Anthocyanins usually produce flowers and fruits in plants with different colors (Cruz et al., 2022); flavanols are mainly enriched in various fruits, such as apples, apricots and plums; and dihydroflavones are mainly present in citrus fruits, making them bitter. Chalcone is a precursor of flavonoids and isoflavones and is widely found in the Faba, Moraceae and ginger families (Ouyang et al., 2021). With the development and improvement of metabonomics techniques, flavonoids from *Vitis vinifera* (De Freitas Laiber Pascoal et al., 2022), *Pyrus communis* (Fischer et al., 2007), *Ficus carica* (Wang et al., 2021), *Fragaria nilgerrensis×pentaphylla* (Shen et al., 2020), *Citrus reticulata×Poncirus trifoliata* (Mou et al., 2021), *Arachis hypogaea* (Wan et al., 2020) and other plants have been studied by previous authors. The biosynthesis of plant flavonoids is specific, and the biosynthesis process for flavonoids and related gene expression are different in different plant varieties, developmental stages, tissues and organs (Wan et al., 2020). To date, flavonoids have been extracted and identified from *Malus pumila* (Henry-Kirk et al., 2012) and *Ziziphus jujuba* (Li et al., 2021), and the components of flavonoids identified in different growth stages of apple and jujube are significantly different. Miguel et al. (2008) determined that the main flavonoids in *Prunus armeniaca* are flavanols, anthocyanins and flavonols, and the contents of flavanols such as gallic acid, catechin and epicatechin are high (Dragovic-Uzelac et al., 2005; Dragovicuzelac et al., 2007; Göttingerová et al., 2021). To date, studies on flavonoids in apricot fruits have been limited to the content of one or more substances and a systematic analysis of their flavonoid metabolites is lacking.

The flavonoid biosynthesis pathway can be divided into the flavonoid and flavonol biosynthesis pathway, the isoflavone biosynthesis pathway and the anthocyanin biosynthesis pathway based on the product formation process (Liu et al., 2021a). There are many related enzymes involved in the biosynthesis of flavonoid substances in the different pathways, and these enzymes generally include phenylalanine ammonia-lyase (PAL), cinnamate 4-hydroxylase (C4H) (Kim et al., 2021), 4-coumarate-CoA ligase (4CL), chalcone synthase (CHS), chalcone isomerase (CHI), naringenin 3-dioxygenase (F3H), flavonol synthase (FLS), bifunctional dihydroflavonol 4-reductase (DFR), UDP-glucose: flavonol 3-O-glucosyltransferase (UFGT), anthocyanidin synthase (ANS), anthocyanidin reductase (ANR), and leucoanthocyanidin reductase (LAR) (Yonekura-Sakakibara et al., 2019; Shen et al., 2022). Several key structural genes involved in flavonoid biosynthesis have been identified in plants such as *Cucumis melo* (Zhang et al., 2021), *Z. jujuba* (Zhang et al., 2020), and *F. nilgerrensis × pentaphylla* (Shen et al., 2020). Song et al. (Song et al., 2022) studied the mechanism of flavonoid biosynthesis in *Camellia oleifera* seeds and found that flavonoid biosynthesis is regulated by genes such as *MYC2*, *bHLH3*, *bHLH18*, *MYB44*, *MYB86*, *WRKY26*, and *WRKY32*. Simultaneously, genetic and molecular studies have shown that structural gene transcription of flavonoid biosynthesis is regulated by ternary transcriptional activation of MBW complexes (Meng et al., 2019). In *Actinidia chinensis*, *Ac*MYBF110-*Ac*bHLH1-*Ac*WDR1 is the main complex that functions (Liu et al., 2021c), while in *M. sieversii*, the main functioning transcription factors are *MdMYB15L* and *MdbHLH33* (Xu et al., 2017; Xu et al., 2018; Xu et al., 2020). Li et al. (Li et al., 2020) studied the effect of transcription factors on red-skinned *P. communis* cv. ‘Starkrimson’ and identified two WRKY transcription factors, *WRKY26* and *WRKY31*, which act together on the MYB promoter, thereby affecting anthocyanin biosynthesis and transport. Plants are mainly regulated by structural genes in the flavonoid biosynthesis pathway, and regulatory genes such as transcription factors mainly affect the synthesis of flavonoid substances by influencing structural genes, but the role of regulatory genes should not be underestimated (Liu et al., 2021b; Ye et al., 2019). Xi et al. (Xi et al., 2019) studied the MYB transcription factors involved in anthocyanin regulation in apricot fruits and determined that *PaMYB10* is a positive regulator of anthocyanin synthesis; in addition, researchers revealed the red skin mechanism of apricot fruits. To date, however, research on the biosynthesis of flavonoids in apricot fruits has not been reported.

Apricot belongs to the Rosaceae, *Prunus*, *Prunophora* Focke, and *Armeniaca* (Lam.) Koch. Native to Xinjiang, the apricot tree is among the oldest fruit trees in China, is an important economic fruit tree species and is distributed throughout China. Xinjiang apricot is rich in germplasm resources, and there are many varieties with different colors, including white, yellow, orange and red; furthermore, its fruit is rich in bioactive compounds that are beneficial to human health (Dragovicuzelac et al., 2007; Hegedus A and Papp, 2011; Sharma S and Thakur, 2017), increasing its value as a functional food. Apricot trees occupy a unique position among drupe fruit trees due to their diverse composition and significant functional potential. Although flavonoid metabolites and related transcription factors in apricot fruit have been studied (Dragovicuzelac et al., 2007; Xi et al., 2019; Göttingerová et al., 2021), the molecular mechanism of flavonoid accumulation and biosynthesis in apricot fruit remains unclear. In this study, the biosynthetic pathway of apricot fruit and the enzyme-encoding genes and transcription factors in the flavonoid biosynthesis pathway were studied by using the combined analysis of metabolome and transcriptome data. Our research provides new insights into apricot flavonoid biosynthesis and molecular breeding.

## Materials and methods

### Plant materials and treatments

**Figure 1:**
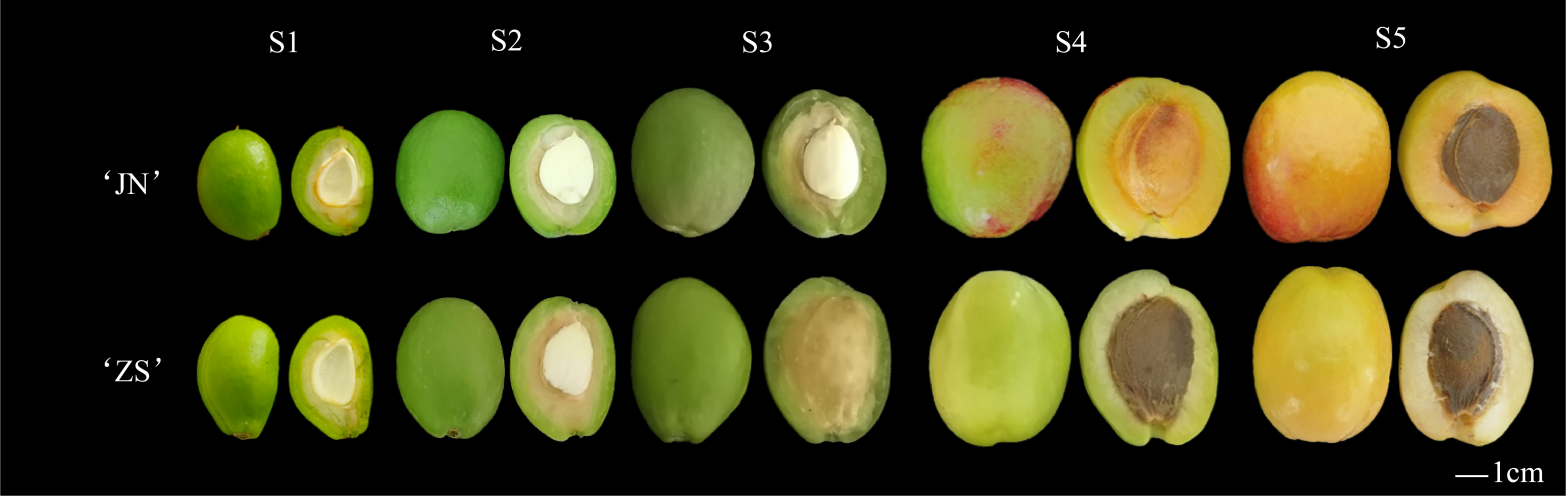
Morphological map of the five reproductive stages of the two apricot cultivars. ‘JN’, *P. armeniaca* cv. ‘Jianali’; ‘ZS’, *P. armeniaca* cv. ‘Zaoshuheiyexing’.

In this experiment, two varieties of apricots were used as test material, namely *P. armeniaca* cv. ‘Jianali’, (JN) (orange flesh) and *P. armeniaca* cv. ‘Zaoshuheiyexing’, (ZS) (white flesh) (Zhou et al., 2021), which were both provided by the Luntai Fruit Tree Resource Nursery, Xinjiang Academy of Agricultural Sciences(E84° 13’, N41° 47’). Apricot fruit from the young (S1) (15 days after flowering), sclerotia (S2) (30 days after flowering), swelling (S3) (50 days after flowering), color change (S4) (70 days after flowering), and finishing (S5) (90 days after flowering) stages were peeled (**Figure 1**) and rapidly stored in liquid nitrogen for later metabolite detection and transcriptome sequencing.

### Extraction identification, analysis of metabolites and screening of differentially accumulated metabolites

#### Sample preparation and extraction

Three biological replicates were established for each sample Biological samples were freeze-dried by a vacuum freeze-dryer (Scientz-100F). The freeze-dried sample was crushed using a mixer mill (MM 400, Retsch) with a zirconia bead for 1.5 min at 30 Hz. Lyophilized powder (100 mg) was dissolved with 1.2 mL 70% methanol solution, vortexed for 30 s every 30 min 6 times in total, and placed in a refrigerator at 4°C overnight. Following centrifugation at 12,000 rpm for 10 min, the extracts were filtered (SCAA-104, 0.22 μm pore size; ANPEL, Shanghai, China, http://www.anpel.com.cn/) before UPLC-MS/MS analysis.

#### UPLC-ESI-MS/MS analysis of metabolites

The sample extracts were analyzed using a UPLC–ESI–MS/MS system (UPLC, SHIMADZU Nexera X2, https://www.shimadzu.com.cn/; MS, Applied Biosystems 4500 Q TRAP, https://www.thermofisher.cn/cn/zh/home/brands/applied-biosystems.html). The analytical conditions were as follows: UPLC: Agilent SB-C18 column (1.8 µm, 2.1 mm × 100 mm); mobile phase consisting of solvent A, pure water with 0.1% formic acid, and solvent B, acetonitrile with 0.1% formic acid. Sample measurements were performed with a gradient program with starting conditions of 95% A and 5% B. Within the first 9 min, a linear gradient to 5% A and 95% B was programmed, and a composition of 5% A and 95% B was maintained for 1 min. Subsequently, a composition of 95% A and 5.0% B was adjusted within 1.1 min and maintained for 2.9 min. The flow velocity was set as 0.35 mL per minute; the column oven was set to 40°C; and the injection volume was 4 μL. The effluent was alternatively connected to an electrospray ionization (ESI)-triple quadrupole (QQQ)-linear ion trap (LIT) (QTRAP)-MS.

The mass spectrometry analysis followed the method by Chen et al. (Chen et al., 2013). The ESI source operation parameters were as follows: ion source, turbo spray; source temperature 550°C; ion spray voltage (IS) 5500 V (positive ion mode)/-4500 V (negative ion mode); ion source gas I (GSI), gas II(GSII), and curtain gas (CUR) were set at 50, 60, and 25.0 psi, respectively; the collision-activated dissociation(CAD) was high. Instrument tuning and mass calibration were performed with 10 and 100 μmol/L polypropylene glycol solutions in QQQ and LIT modes, respectively. QQQ scans were acquired as multiple reaction monitoring (MRM) experiments with collision gas (nitrogen) set to medium. DP and CE for individual MRM transitions were performed with further DP and CE optimization. A specific set of MRM transitions was monitored for each period according to the metabolites eluted within this period.

#### Qualitative and quantitative analysis of metabolites

The qualitative and quantitative analysis of metabolites followed the method by Wang et al. (Wang et al., 2018). Based on a self-built database called MWDB (Metware database), the substances were characterized utilizing secondary spectral information, and the analysis was performed by removing isotopic signals, duplicate signals containing K^+^ ions, Na^+^ ions, NH^4+^ ions, and duplicate signals of fragment ions that are other larger molecular weight substances. The raw mass spectrometry data were imported into Analyst 1.6.3, and Analyst software was used to process the mass spectrometry data. Metabolites were annotated by a local database, and metabolites were quantified using MRM analysis of QQQ mass spectra. The extraction, detection, and quantitative analysis of metabolites in the samples were performed by Wuhan Metware Biotechnology Co., Ltd. (www.metware.cn).

#### Principal component analysis

Unsupervised principal component analysis (PCA) was performed by the statistics function prcomp within R (www.r-project.org). The data were unit variance scaled before unsupervised PCA.

#### Hierarchical cluster analysis and pearson correlation coefficients

The hierarchical cluster analysis (HCA) results of samples and metabolites are presented as heatmaps with dendrograms, while Pearson correlation coefficients (PCCs) between samples were calculated by the cor function in R and are presented as only heatmaps. Both HCA and PCC calculation were carried out by the R package heatmap. For HCA, normalized signal intensities of metabolites (unit variance scaling) are visualized as a color spectrum.

#### Screening for differentially accumulated metabolites

Based on the orthogonal projections to latent structures discriminant analysis (OPLS-DA results) and the fold change, the metabolites of variable importance in projection (VIP) ≥ 1 and |log_2_Fold Change| ≥ 1 were selected from the obtained VIP of the multivariate analysis OPLS-DA model, and metabolites with VIP ≥ 1 and |log_2_Fold Change| ≥ 1 were initially screened for differences between species. The VIP value indicates the intensity of the influence of the intergroup differences of the corresponding metabolites in the model in the classification discriminations of each group of samples, and metabolites with VIP ≥ 1 are generally considered significant differences.

### Transcriptome sequencing

RNA was extracted from the pulp of two apricot varieties at different stages of fertility using a polysaccharide polyphenol RNA extraction kit, and the RNA quality and concentration were determined by agarose gel electrophoresis, OD260/280 ratio and A260/A230 ratio. The starting RNA was total RNA, ≥1 μg, and the library kit used was Illumina’s NEBNext^®^UltraTM RNA Library Prep Kit. mRNA with polyA tails was enriched by oligo (dT) magnetic beads, and the resulting mRNA was subsequently randomly interrupted with divalent cations in NEB Fragmentation Buffer. The fragmented mRNA was used as a template and random oligonucleotides as primers to synthesize the first strand of cDNA in the M-MuLV reverse transcriptase system, followed by degradation of the RNA strand with RNaseH and synthesis of the second strand of cDNA with dNTPs under the DNA polymerase I system. The purified double-stranded cDNA was end-repaired, and after end-repair, an A-tail was added. Sequence connectors were ligated and cDNA of approximately 200 bp was screened with AMPure XP beads. PCR amplification was performed, and the PCR products were purified again with AMPure XP beads to finally obtain the library. After the library was constructed, the library was initially quantified using a Qubit 2.0 Fluorometer and diluted to 1.5 ng/µL. Then, the insert size of the library was determined using an Agilent 2100 bioanalyzer. After the insert size met expectations, the effective concentration of the library was accurately quantified by qRT-PCR (effective library concentration above 2 nM).

After checking the library, the different libraries were pooled according to the effective concentration and the target downstream data volume required for Illumina sequencing, and 150 bp paired-end reads were generated.

### Transcriptome analysis

#### Data quality control

The raw data were filtered using fastp 0.19.3 to remove reads with connectors; all subsequent analyses were based on clean reads.

#### Sequence alignment to the reference genome

The reference genome (*P. armeniaca* Longwangmao Whole Genome v1.0 Assembly & Annotation) and its annotation file were downloaded from GDR (Jung et al., 2018) and indexed using HISAT 2.1.0, and clean reads were compared to the reference genome.

#### Quantification of gene expression levels and screening of differentially expressed genes

The gene comparisons were calculated using FeatureCounts 1.6.2, and then the fragments per kilobase per million mapped fragments (FPKM) was calculated for each gene based on the gene length. FPKM is currently the most commonly used method to estimate gene expression levels. Differential expression analysis between the two groups was performed using DESeq2 1.22.1, corrected for *P values* using the Benjamini & Hochberg method. Corrected *P values* < 0.05 and |log_2_Fold Change| ≥ 1 were used as the thresholds for significant differential expression.

#### Differentially expressed gene enrichment analysis

Enrichment analysis was performed based on a hypergeometric test, and for Kyoto Encyclopedia of Genes and Genomes (KEGG) analysis, a hypergeometric distribution test was performed in Pathway; differentially expressed genes (DEGs) were enriched in the plant flavonoid biosynthesis pathway (Pathway KO00941).

### Weighted gene coexpression network analysis (WGCNA)

The WGCNA package in R language was used to construct a coexpression network of genes, and all screened differentially accumulated metabolites and DEGs were imported into WGCNA. All genes were divided into different modules according to the expression pattern, and the genes in the same module were absolutely related. The module eigengene value of each module is the first principal component of the module and can represent the gene expression pattern in the module. The correlation and significance of gene matrix values and differentially accumulated metabolites were calculated by the Significance function inside the R language WGCNA package to determine the core modules of differentially accumulated metabolites, and the first twenty core genes of the core module were analyzed with the differentially accumulated metabolites using the correlation network tool. The results obtained from the analysis were imported into Cytoscape software for visual display.

### Quantitative real time-PCR validation

Ten key genes in the KEGG pathway were selected and the transcriptome data were validated by qRT-PCR. The actin and cyclophilin genes of apricot were used as internal reference controls (Niu et al., 2014), primers (**Supplementary Table 1**) were added and PCR amplification was performed. The relative expression levels of the genes were determined using the cyclic threshold method 2^-ΔΔCT^. Three biological replicates were set for each treatment, and each biological replicate was then subjected to three technical replicates. RNA was extracted from apricot pulp using a plant polysaccharide polyphenol RNA extraction kit, and first-strand cDNA was obtained using a cDNA reverse transcription kit. Primers were designed using the online software Primer 3 Plus, and after design, they were checked using the software DNAMAN to confirm that no primers between the primers were designed using the online software Primer 3 Plus.

## Results

### Differentially accumulated metabolite analysis

To analyze the differences in flavonoid metabolites of JN and ZS at different periods, flavonoid metabolites were detected using UPLC-MS/MS. The total ion flow (TIC) plots obtained in QC samples reflect the sum of all ion intensities at different time points (**Supplementary Figure 1**). In the detection multipeak plot (XIC), peaks of different colors correspond to different species of flavonoid metabolites (**Supplementary Figure 2**). The overlay of the TIC plots of QC samples showed that the response intensities and retention times of each peak strongly overlapped, indicating that the data were reliable (**Supplementary Figure 3**). A total of 222 flavonoids were found in JN and ZS fruits at different periods (**Supplementary Table 2**), and these flavonoids were classified into 11 classes, including flavonols (32.9%), flavones (16.6%), anthocyanidins (9.0%), tannins (8.6%), flavanols (8.1%), flavanones (6.7%), chalcones (5.0%), flavanonols (4.5%), proanthocyanidins (4.5%), flavonoid carbonosides (2.3%), and isoflavones (1.8%) (**Supplementary Figure 5C**). The relative contents of flavonoids in ‘JN’ and ‘ZS’ at different periods were analyzed by clustering (**Supplementary Figure 4**), and the results showed that there were significant differences in the accumulation patterns of flavonoids in JN and ZS in different periods, among which the relative content of accumulated flavonoids in JNS1 and ZSS1 was higher. Notably, ZS contains three specific flavonoid metabolites, namely syringetin-3-O-rutinoside (flavonol), cyanidin-3-O-(6’’-O-caffeoyl-2’’-O-xylosyl) glucoside (anthocyanin) and petunidin-3-O-(6’’-O-p-coumaroyl) glucoside-5-O-rhamnoside (anthocyanin). The results of principal component and correlation analyses showed that the flavonoid metabolites of JN and ZS showed effective separation at different periods (**Supplementary Figure 5A**) and strong correlation between different replicates (**Supplementary Figure 5B**), which demonstrated that the metabolome data were reproducible and reliable.

**Figure 2:**
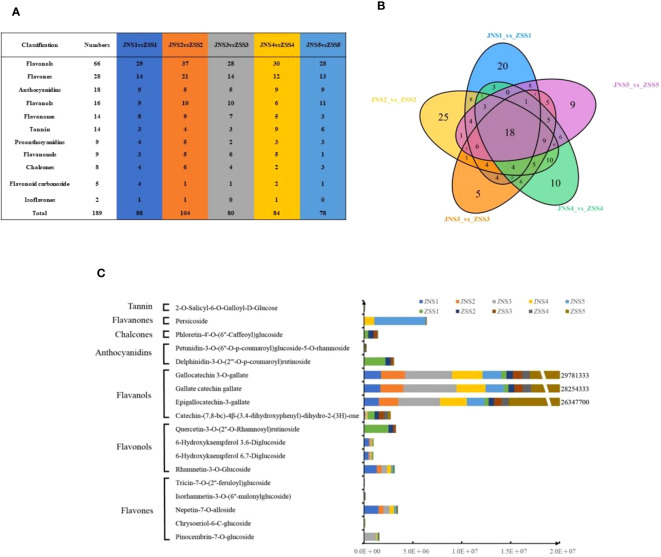
Sampling and variance analysis of two apricot fruits at different fertility stages. **(A)** statistics of differentially accumulated metabolite types in different comparison groups; **(B)** Venn diagram of differentially accumulated metabolites; **(C)** relative contents of 18 differentially accumulated metabolites at different periods in JN and ZS.

Based on the metabolome data, 88, 104, 80, 84, and 78 DAFs were identified between the JNS1 vs. ZSS1, JNS2 vs. ZSS2, JNS3 vs. ZSS3, JNS4 vs. ZSS4, and JNS5 vs. ZSS5 groups, respectively (**Figure 2A**). Through Venn diagram analysis, 18 kinds of shared DAFs were identified in JN and ZS in different periods (**Figure 2B**). Ten DAFs (five flavonoids, three flavonols, one dihydroflavonoid, and one ellagitannin) were significantly changed in JN in multiple periods compared to ZS. Compared with JN, 8 DAFs (1 flavonol, 4 flavanols, 2 anthocyanins, 1 chalcone) were significantly changed in multiple periods in ‘ZS’ (**Figure 2C**). Among them, three flavanols (epigallocatechin-3-gallate, gallate catechin gallate and gallocatechin 3-O-gallate) accumulated significantly in the first four stages of JN; in the fifth period, they accumulated significantly in ZS (the difference multiples were 14.6, 15.2 and 15.4, respectively).

### Transcriptome sequencing, assembly and statistics

**Figure 3:**
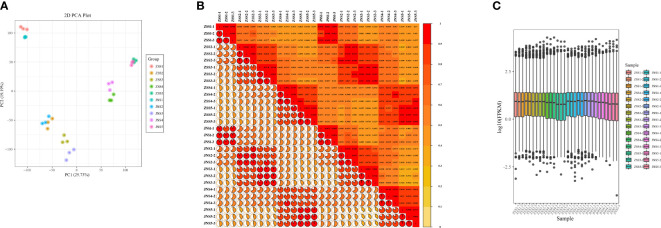
Gene expression and correlation of each sample. **(A)** principal component analysis of each sample; **(B)** correlation coefficient plot of each sample; **(C)** box plot of gene expression levels.

To further investigate the differences in the synthesis mechanism of flavonoids between JN and ZS, we performed *de novo* transcriptome sequencing at different periods (30 samples). By high-throughput sequencing, 43156846~61327158 raw sequences (raw reads) were obtained for each sample; after removing the connectors and low-quality sequences, 40,673,760~57,231,530 sequences (clean reads) were obtained for each sample. The distribution of Q30 bases in the clean reads was over 92.43%, with an average GC content of 45.8% (**Supplementary Table 3**). The transcriptome data were compared with the reference genome, and all samples were matched at more than 93% (**Supplementary Table 4**). The PCA plots (**Figure 3A**), correlation matrix (**Figure 3B**) and gene expression levels (**Figure 3C**) were analyzed and the correlation between the samples was high and met the criteria for transcriptomic analysis.

### Expression analysis of differentially expressed genes and KEGG enrichment

**Figure 4:**
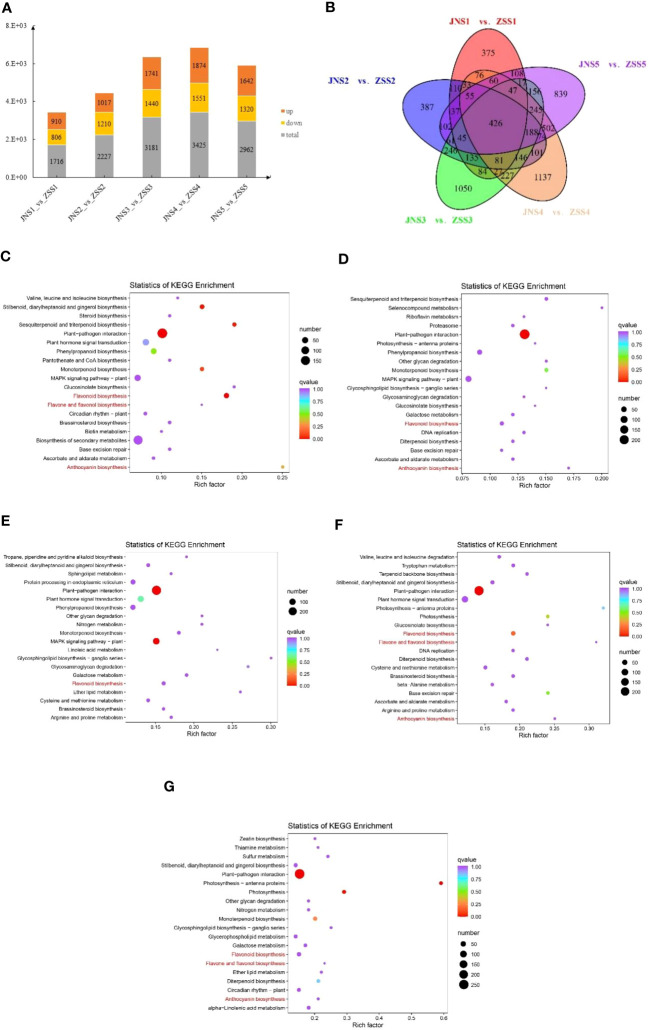
Analysis of the differentially expressed genes in JN and ZS. **(A)** statistics of DEGs in different JN and ZS comparison groups, orange indicates upregulation, yellow indicates downregulation, and gray indicates total; **(B)** Venn diagram of DEGs; **(C-G)**, bubble plots of DEGs enriched KEGG between the five JN and ZS comparison groups; **(C)** JNS1 vs. ZSS1; **(D)** JNS2 vs. ZSS2; **(E)** JNS3 vs. ZSS3; **(F)** JNS4 vs. ZSS4; **(G)** JNS5 vs. ZSS5.

We used DESeq2 software to analyze 30 samples and obtained 15,856 DEGs. There were 1716, 2227, 3181, 3181, and 2962 DEGs between the JNS1 vs. ZSS1, JNS2 vs. ZSS2, JNS3 vs. ZSS3, JNS4 vs. ZSS4, and JNS5 vs. ZSS5 comparison groups, respectively. There were 3425 and 2962 DEGs, of which 910, 1017, 1741, 1874, and 1642 were upregulated DEGs and 806, 1210, 1440, 1551, and 1320 were downregulated DEGs, respectively (**Figure 4A**). Compared with the genes expressed in ZS, JNS4 contained the most up- and downregulated genes, indicating that this period is a critical period for different fertility stages. The results of Venn diagram analysis showed that a total of 426 DEGs were expressed among the five comparison groups (**Figure 4B**), indicating that these DEGs may play a key role in flavonoid biosynthesis in JN and ZS.

The DEGs identified between the JN and ZS comparison groups involved a total of 136 KEGG metabolic pathways, and 36, 34, 39, 31, and 35 of these metabolic pathways were significantly enriched (*q* value > 0.9) between the JNS1 vs. ZSS1, JNS2 vs. ZSS2, JNS3 vs. ZSS3, JNS4 vs. ZSS4, and JNS5 vs. ZSS5 comparison groups, respectively. Among them, flavonoid biosynthesis (ko00941) was significantly enriched in the five comparison groups. Anthocyanin biosynthesis (ko00942) was significantly enriched in the JNS1 vs. ZSS1, JNS2 vs. ZSS2, JNS4 vs. ZSS4, and JNS5 vs. ZSS5 comparison groups, and flavonoid and flavonol biosynthesis (ko00944) was significantly enriched in the JNS1 vs. ZSS1, JNS4 vs. ZSS4, and JNS5 vs. ZSS5 comparison groups (**Figures 4A–G**).

### Trend clustering analysis for the differentially expressed genes

To understand the expression patterns of DEGs at different times in JN and ZS, the expression of all DEGs (fragments per kilobase million, FPKM) was clustered by K-means (**Supplementary Figure 6**). All DEGs were divided into 16 categories, the expression of DEGs in each category was consistent, and it was presumed that the exercise functions of the DEGs were similar.

### Identification of the key genes for flavonoid biosynthesis and pathway mapping

**Figure 5:**
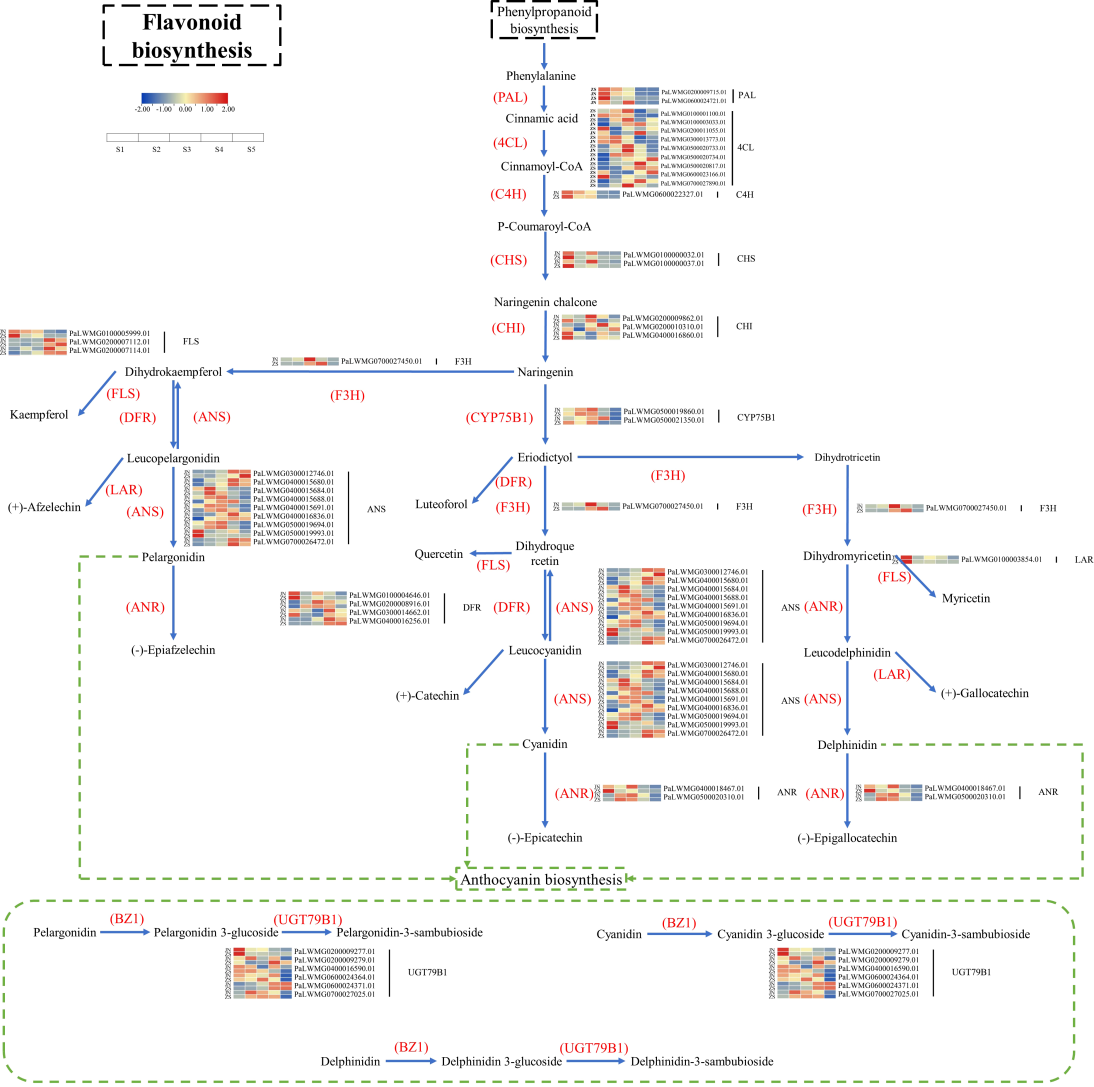
Diagram of the flavonoid biosynthesis pathway in apricot fruit.

Based on KEGG enrichment analysis and gene function annotated DEGs, a total of 70 structural genes (at least one period FPKM>10) regulating 17 enzymes were identified, namely, PAL, 4CL, C4H, HCT, C3’H, CHS, CHI, F3H, F3’H (CYP75B1), F3’5’H, FLS, DFR, LAR, ANS, ANR, UGT79B1, CYP81E, including 34 genes regulating 11 enzymes of flavonoid biosynthesis, anthocyanin biosynthesis, flavonoid and flavonol biosynthesis (**Supplementary Table 5**). Based on the 34 key enzyme-encoding genes for flavonoid biosynthesis and the KEGG synthesis pathway (ko00941), the flavonoid biosynthesis pathway of apricot fruit was mapped (**Figure 5**). The 11 key enzymes were C4H (cinnamic acid 4-hydroxylase [EC 1.14.14.91]), CHS (chalcone synthase [EC:2.3.1.74]), CHI (chalcone isomerase [EC:5.5.1.6]), F3H (naringenin 3-dioxygenase [EC:1.14.11.9]), FLS (flavonol synthase [EC:1.14.20.6]), DFR (bifunctional dihydroflavonol 4-reductase/flavanone 4-reductase [EC:1.1.1.219 1.1.1.234]), ANS (anthocyanidin synthase [EC:1.14.20.4]), CYP75B1 (flavonoid 3’-monooxygenase [EC:1.14.14.82]), ANR (anthocyanidin reductase [EC:1.3.1.77]), LAR (leucoanthocyanidin reductase [EC:1.17.1.3]), and UGT79B1 (anthocyanidin 3-O-glucoside 2’’’-O-xylosyltransferase [EC:2.4.2.51]). Among the 34 enzyme-encoding genes that regulate flavonoid biosynthesis in apricot fruit, *CYP75B12* and *ANS9* were highly expressed at ZSS3 and ZSS4 with FPKM values of 367.49 and 272.81, respectively, and *CYP75B12* and *ANS9* were highly expressed at JNS4 with FPKM values of 362.10 and 336.04, respectively. In addition, one specific enzyme-encoding gene (*UGT79B12*) was found to be significantly accumulated in different periods in ‘ZS’.

**Figure 6:**
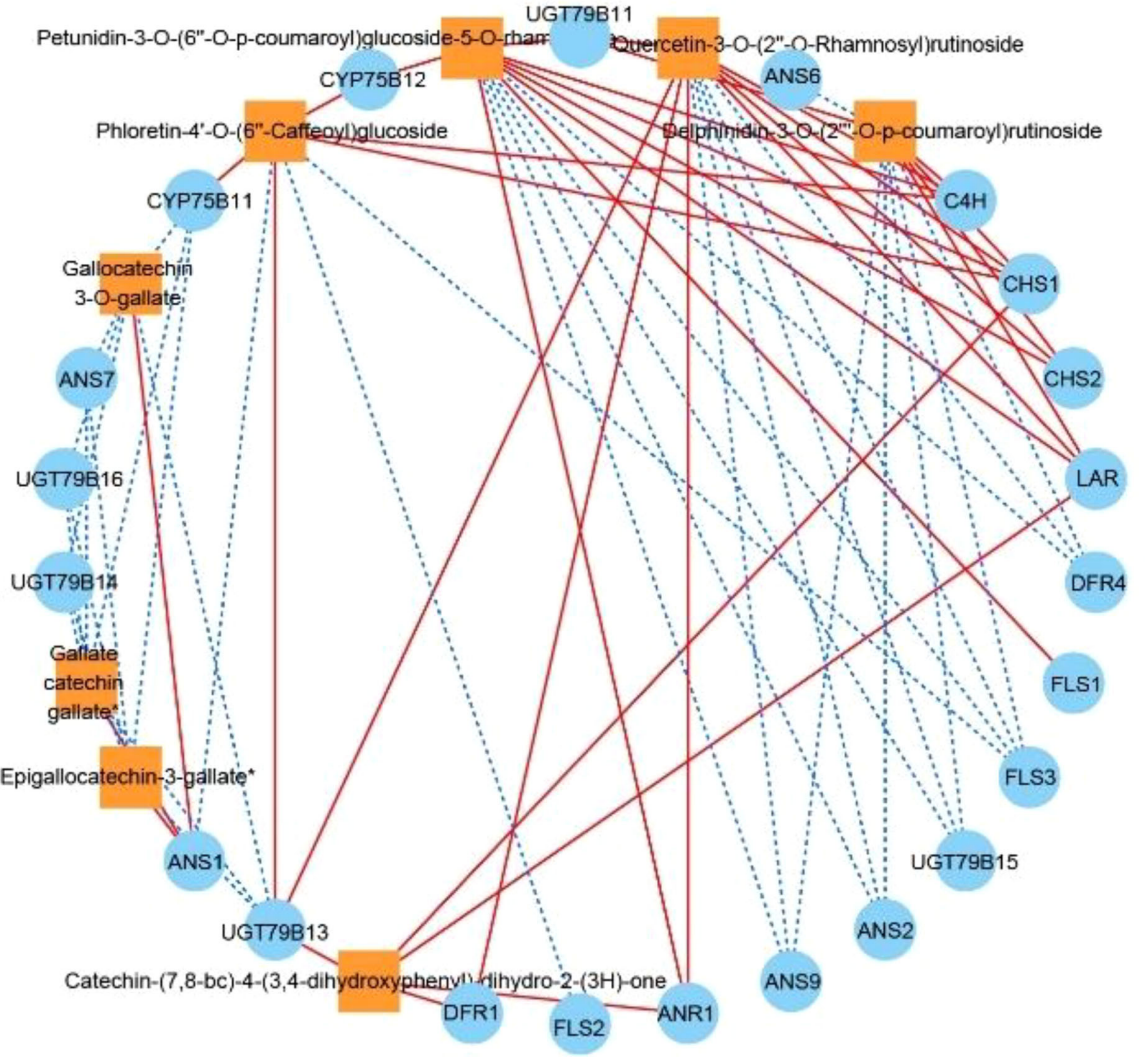
Correlation network diagram of eight DAFs in ZS and key structural genes involved in flavonoid biosynthesis. Orange boxes represent DAFs, blue circles represent enzyme-encoding genes, red solid lines represent a positive correlation between the two, and blue dashed lines represent a negative correlation between the two.

We subjected each of the 34 structural genes to correlation network analysis (threshold > 0.8) with the eight DAFs that were significantly accumulated in ZS (**Figure 6**), and the results showed that the DAFs were significantly correlated with 22 structural genes. Among them, catechin-(7,8-bc)-4β-(3,4-dihydroxyphenyl)-dihydro-2-(3H)-one was positively correlated with all structural genes. Nine structural genes were positively and negatively correlated with DAFs; furthermore, correlation network analysis was performed at a threshold >0.96, and the results showed that *C4H*, *CHS1*, and *CHS2* were positively correlated with petunidin-3-O-(6’’-O-p-coumaroyl) glucoside-5-O-rhamnoside, while *ANS9* was negatively correlated with the compound. *UGT79B11* was positively correlated with delphinidin-3-O-(2’’’-O-p-coumaroyl) rutinoside, and *FLS3*, *DFR4*, *UGT79B15*, *ANS2* and *ANS9* were negatively correlated with the compound. *FLS3*, *DFR4* and *UGT79B15* were negatively correlated with quercetin-3-O-(2’’-O-rhamnosyl) rutinoside. *FLS2* was negatively correlated with phloretin-4’-O-(6’’-caffeoyl) glucoside. *DFR1* was positively correlated with catechin-(7,8-bc)-4β-(3,4-dihydroxyphenyl)-dihydro-2-(3h)-one. Based on the above analysis, there were 11 DEGs that were highly correlated with 5 DAFs, and we speculate that these 11 DEGs are likely involved in regulating the biosynthesis of these 5 DAFs.

### Transcription factor-mediated analysis of flavonoid biosynthesis

**Figure 7:**
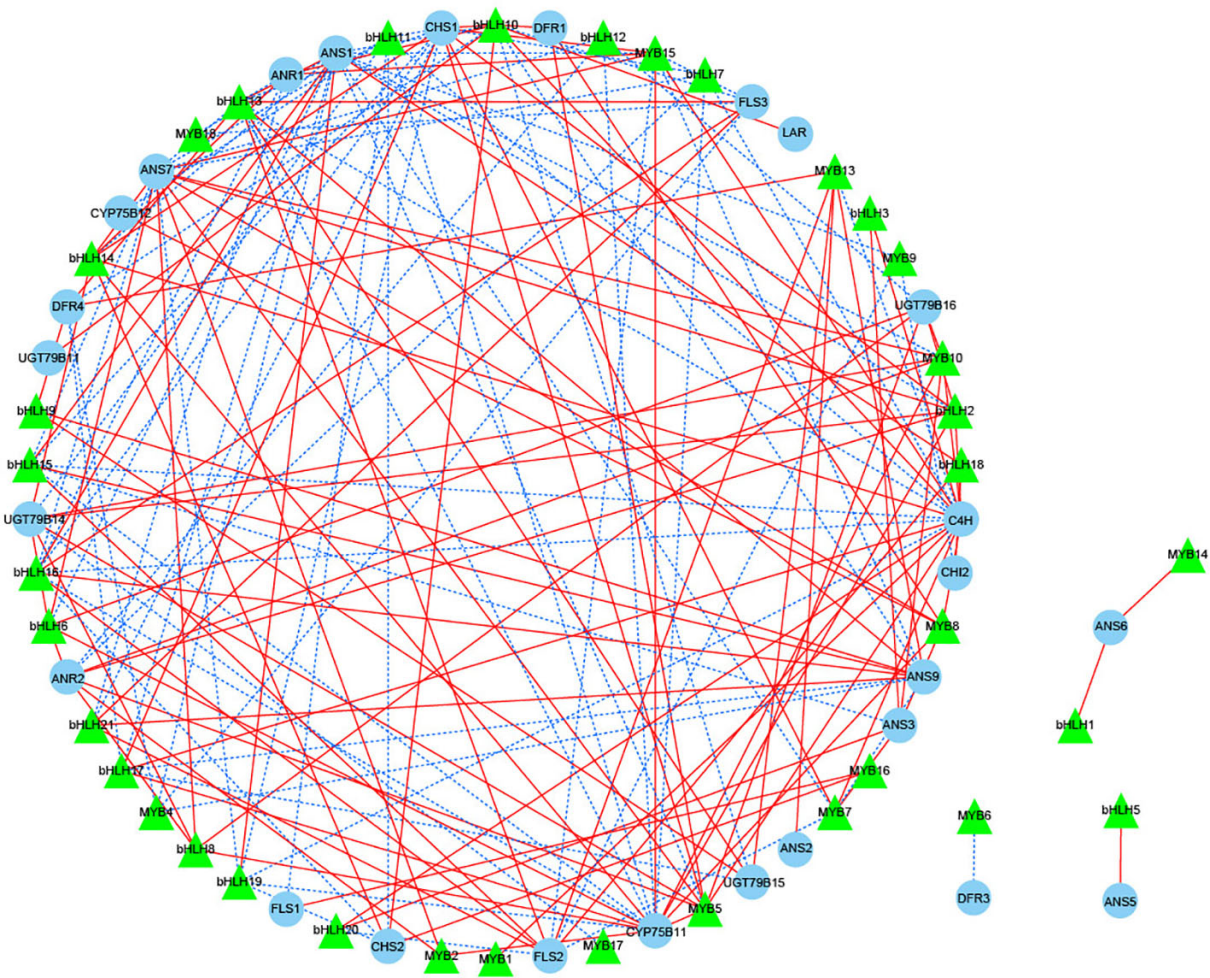
Correlation network diagram of key structural genes involved in flavonoid biosynthesis with MYB and bHLH transcription factors. Green triangles represent transcription factors, blue circles represent key structural genes involved in flavonoid biosynthesis, red solid lines represent a positive correlation between the two, and blue dashed lines represent a negative correlation between the two.

The flavonoid biosynthetic pathway is regulated by both structural genes and transcription factors (TFs), and in the present study, the most numerous families of TFs were identified in different periods of JN and ZS (Top 8 classes), including MYB, NAC, AP2/ERF-ERF, bHLH, C2H2, C3H, WRKY, B3, etc. We screened 18 MYBs and 21 bHLHs (one or more periods with FPKM values > 20) that were associated with flavonoid synthesis (**Table S6**) to perform correlation analysis with 34 structural genes of flavonoid biosynthesis (threshold value > 0.8) (**Figure 7**). The results showed that 15 MYBs and 20 bHLHs were significantly correlated with 26 structural genes for flavonoid biosynthesis (*P* < 0.05). *DFR3* was negatively correlated with *MYB6*, *ANS5* was positively correlated with *bHLH5*, and *ANS6* was positively correlated with both MYB14 and bHLH1; we further performed correlation analysis for the threshold value > 0.98, and the results showed that *ANS7* was positively correlated with *bHLH8*, and *CYP75B11* was also positively correlated with *bHLH2* and *MYB5*. Based on the above analysis, we speculate that *bHLH2*, *bHLH8* and *MYB5* likely interact with the structural genes of flavonoid biosynthesis, which in turn regulates the synthesis of flavonoid substances.

### Weighted gene coexpression network analysis

**Figure 8:**
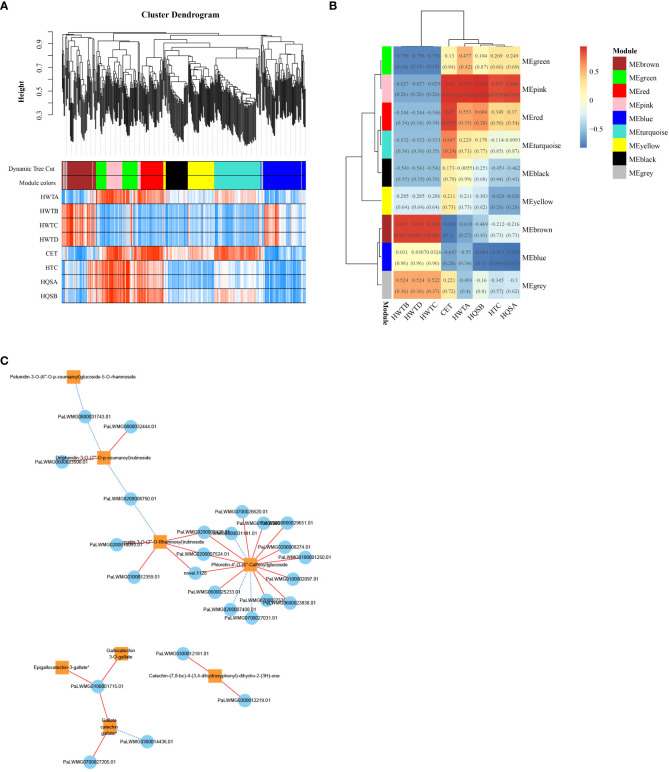
ZS differentially expressed genes and WGCNA results for differentially accumulated metabolites. **(A)** clustering dendrogram results showing 9 expression modules, each marked with a different color; **(B)** 8 DAF correlation analysis modules in ‘ZS’, the value in each box indicates the Pearson correlation coefficient between the modules containing DAFs, and the number in each bracket indicates the *P* value. The color scale on the right side represents the correlation degree between the modules and DAFs, and the red color represents high correlation; **(C)** Correlation network diagram of DEGs and 8 DAFs. The orange box indicates DAFs, the blue circle indicates DEGs, the red solid line indicates positive regulation between the two, and the blue dashed line indicates negative regulation between the two.

To investigate the gene regulatory network (weighted gene coexpression network analysis, WGCNA) in the flavonoid biosynthetic pathway of ZS, the FPKM values of overlapping DEGs (426 DEGs with a common overlap among the five comparison groups) and DAFs (8 DAFs significantly accumulated in ZS) were used as source data for WGCNA. A total of nine DEG modules were identified in the clustering dendrogram (**Figure 8A**), and three of them, MEpink, MEbrown, and MEred, were highly correlated with the contents of the eight DAFs mentioned above (**Figure 8B**). In addition, correlation analysis was performed between the hub genes (top 20 DEGs) in the modules MEpink (weight > 6.35), MEbrown (weight > 7.76) and MEred (weight > 10.71) and the 8 DAFs. In the module, 26 DEGs (threshold > 0.95, *P* value < 0.01) were selected to construct the correlation network, including 15 enzyme-encoding genes, 8 protein-related genes and 3 genes of unknown function.

These genes all positively regulated one or more DAFs (**Figure 8C**), in which phloretin-4’-O-(6’’-caffeoyl) glucoside was the core of the network graph, which was positively regulated by 12 genes. This was followed by quercetin-3-O-(2’’-O-Rhamnosyl) rutinoside which was positively regulated by 5 genes, followed by delphinidin-3-O-(2’’’-O-p-coumaroyl) rutinoside and catechin-(7,8-bc)-4β-(3,4-dihydroxyphenyl)-dihydro-2-(3h)-one which were positively regulated by 2 genes each. Notably, the analysis of the previous metabolites showed that the three flavanols with great differences were coregulated by one gene. The results suggest that these 26 genes play an important regulatory role in the flavonoid biosynthetic pathway of ZS.

### Quantitative real time-PCR validation

To verify the accuracy of the transcriptome data, 10 DEGs in the regulatory flavonoid metabolic pathway were selected and validated using real-time fluorescence quantification (Qrt-PCR). The results showed that the qRT-PCR gene expression trends were generally consistent with the transcriptome expression trends, indicating that the transcriptome data were valid and reliable (**Supplementary Figure 7**).

## Discussion

### Analysis of the flavonoid compounds in apricot fruits

Flavonoid compounds have extremely strong medicinal value for humans, and studies on flavonoid compounds in apricot fruit have focused on determining the total flavonoid content and analyzing the species and contents of anthocyanins (Dragovic-Uzelac et al., 2005; Dragovicuzelac et al., 2007; Xi et al., 2019; Göttingerová et al., 2021). In the present study, 222 flavonoid compounds were identified by LC-MS/MS in five periods of JN and ZS, and these metabolites were mainly flavonols and flavones, followed by anthocyanins, ellagitannins and flavanols. In the groups comparing JN and ZS at different periods (**Figures 2B, C**), 18 DAFs were found. These DAFs were found in JN and ZS and showed significant differences in the accumulation patterns, with eight DAFs significantly accumulating in ZS. Notably, three flavanols were significantly more abundant in ZS than in JN. Verica et al. (Dragovicuzelac et al., 2007) investigated polyphenols in apricot fruit and showed that gallic acid esters, (+)-catechin and (-)-epi catechins were the substances with the highest content in apricot fruits, and the results of this study were basically consistent with these results. In addition, the results of this study showed that apricot fruit flavonoids accumulated significantly in the young fruit stage, especially flavonol compounds, which was consistent with the trend of flavonoids in apples (Henry-Kirk et al., 2012).

### Analysis of the enzymes and key structural genes related to flavonoid biosynthesis in apricot fruits

Recently, combined transcriptomic and metabolomic analysis has become an analytical approach to determine the relationship between genes and metabolites related to the biosynthetic pathway of a substance in a species (Wu et al., 2022; Yuan et al., 2022). In this study, based on the plant flavonoid biosynthetic pathway (Pathway ko00941) and transcriptome data for apricot fruit flesh, we obtained an apricot fruit flavonoid biosynthetic pathway map using combined transcriptomic and metabolomic analysis, and identified 11 key enzymes (C4H, CHS, CHI, F3H, FLS, CYP75B1, DFR, ANS, ANR, LAR and UGT79B1), and 34 genes regulating key enzymes were also screened. These genes were similar to those found for flavonoid biosynthesis in *C. melo* (Zhang et al., 2021) pericarp, but *4CL*, *IFS*, *FNS*, and *UFGT* were not present in the flavonoid biosynthesis pathways of the involved apricot fruits, probably due to interspecies differences. To uncover key structural genes highly correlated with flavonoid biosynthesis, we hypothesized that DEGs are positively regulated when the change trends are consistent with those of differentially accumulated flavonoid (DAF) metabolites, and genes exhibiting trends opposite to their change trends are negatively regulated genes. Based on the correlation analysis between metabolites and structural genes, *C4H*, *CHS1*, *CHS2*, *UGT79B11*, and *DFR1* were positively regulated genes, while *ANS2*, *ANS9*, *FLS2*, *FLS3*, *DFR4*, and *UGT79B15* were negatively regulated genes. Furthermore, this study found that the structural genes associated with catechin-(7,8-bc)-4β-(3,4-dihydroxyphenyl)-dihydro-2-(3h)-one were all positively correlated. When the threshold value was >0.96, *DFR1* was positively correlated with the gene, and we speculated that the accumulation of catechin-(7,8-bc)-4β-(3,4-dihydroxyphenyl)-dihydro-2-(3h)-one might be regulated by *DFR1*. Notably, flavanols similar to the catechin-(7,8-bc)-4β-(3,4-dihydroxyphenyl)-dihydro-2-(3h)-one class play an important role in preventing cardiovascular diseases in humans, as their effects on human health mainly target neurological and cardiovascular diseases (Panche et al., 2016; Joshi et al., 2018). In this study, the preliminary gene regulation pattern of flavonoid biosynthesis in apricot fruit was identified by combined multiomics analysis, and importantly, one key gene regulating the synthesis of catechin-(7,8-bc)-4β-(3,4-dihydroxyphenyl)-dihydro-2-(3h)-one was also screened; thus, a candidate gene was provided for selecting and breeding high-quality apricot varieties in the future.

### Analysis of transcription factors and candidate genes involved in flavonoid biosynthesis in apricot fruits

Plant flavonoid biosynthesis is regulated not only by structural genes but also by the expression levels of MYB, bHLH and WD40 TFs and MYB-bHLH-WD40 (MBW) complexes (Hichri et al., 2011; Meng et al., 2019). In the present study, the highest number of MYB TFs (including MYB-related TFs) was found in apricot fruits at different times, and some NAC, AP2, and bHLH TFs were also identified. Notably, no WD40 TFs were identified in the apricot fruits, suggesting that MYB and bHLH are the main TF families regulating flavonoid biosynthesis in apricot fruits. MYB TFs can be categorized as 1R-MYB (1R-MYB and Myb-related), R2R3-MYB, 3R-MYB and 4R-MYB according to the number of sequences containing highly conserved DNA binding domains (Li et al., 2019). bHLH TFs are named based on their highly conserved structural domains that contain two regions (basic region and HLH region) (Toledo-Ortiz et al., 2003). In many cases, MYB and bHLH interact and thus exert functions on structural genes, such as *A. chinensis AcMYBF110-AcbHLH1-AcWDR1* (Liu et al., 2021c) and *M. sieversii MdMYB15L*, which interact with the activator *MdbHLH33*; these genes work together to regulate changes in anthocyanin production (Xu et al., 2017; Xu et al., 2018; Xu et al., 2020). In the present study, 15 MYB TFs and 20 bHLH TFs were associated with 26 structural genes for flavonoid biosynthesis. When a threshold value > 0.98, *MYB5*, *bHLH2*, and *bHLH8* were found to be positively correlated with *ANS7* and *CYP75B11*, key structural genes for flavonoid biosynthesis, from which we hypothesized that *bHLH8* is positively regulated with *ANS7*; *MYB5* and *bHLH2* interact with each other and are positively regulated with *CYP75B11*, and three TFs found in JN and ZS showed the same expression trend.

To more strongly support the process of flavonoid biosynthesis in apricot fruits, this study identified key genes involved in regulating the accumulation of eight DAFs in ZS by WGCNA, and selected 26 genes with high correlation to construct a correlation network diagram (**Figure 8C**). The results showed that these 8 DAFs were regulated by 1-15 genes. In WGCNA, three genes (*PaLWMG0100001250.01*, *PaLWMG0200007408.01*, *PaLWMG0800029651.01*) simultaneously targeted phloretin-4’-O-(6’’-Caffeoyl) glucoside. This result indicates that these 26 genes play an important regulatory role in the flavonoid biosynthetic pathway in ZS, and their specific functions deserve further investigation. In addition, the transcriptome data were also validated by qRT–PCR, and the results showed that the transcriptome data were consistent with the change trend observed for the qRT–PCR data, which fully confirmed the accuracy of the transcriptome data.

## Conclusion

In summary, we synthetically compared the transcriptomes and metabolic profiles of two apricot cultivars (JN and ZS) at different times, revealed 222 flavonoid metabolites and 15856 DEGs in apricot fruits, and identified 26 candidate genes related to eight DAFs. In addition, we analyzed the apricot fruit flavonoid biosynthesis pathway and obtained 34 structural genes regulating 11 key enzymes and 3 transcription factors associated with 2 structural genes. Our results provide new insights into the regulation of apricot fruit flavonoid biosynthesis and the functional identification of key genes.

## Data availability statement

The datasets presented in this study can be found in online repositories. The names of the repository/repositories and accession number(s) can be found below: https://www.ncbi.nlm.nih.gov/, PRJNA921732.

## Author contributions

YC: Formal analysis, writing - first draft. KL: supervision. LL: investigation, validation. GF: resources, software. SZ: validation, visualization. YW: resources, visualization. KJ: methodology, writing-review and editing, investigation. WL: Writing-review and editing, supervision, funding acquisition. All authors contributed to the article and approved the submitted version.
