# Supplementary material for: Metabolomic and transcriptomice analyses of flavonoid biosynthesis in apricot fruits
Source: Front Plant Sci. 2023 Jul 18;14:1210309. doi: 10.3389/fpls.2023.1210309 (PMC10390783; doi:10.3389/fpls.2023.1210309)
Supplement: Supplementary file 1 [file DataSheet_1.docx]

**Supplemental files**

Table S1 Real-time PCR primer sequences

| Gene ID | Self-serial number | Primer sequence (5'-3') | Dissolving temperature（℃） | |
| --- | --- | --- | --- | --- |
| PaLWMG0200009862.01 | Lht1F | TGCTTTCGGAGGCAGTTCTAG | | 57.2 |
|  | Lht1R | TCCGGCCTCATTATGACAACTC | | 56.9 |
| PaLWMG0200010310.01 | Lht2F | GCAACTTCTGCCACTGCTGAG | | 59.5 |
|  | Lht2R | AGCTGAGAGTGTGTTAGCCAAGG | | 59.3 |
| PaLWMG0400016860.01 | Lht3F | GCAATTTGCACTCGAACAAGGC | | 58.0 |
|  | Lht3R | AAAGAGCAAGAGTGGGTTTGGC | | 58.3 |
| PaLWMG0700027450.01 | Lht4F | GCATTGACAAAGGCGTGTGTTG | | 57.9 |
|  | Lht4R | TTGAACGGTGATCCACGTCTTCC | | 59.6 |
| PaLWMG0200007112.01 | Lht5F | TGTACTTGGGATCGAAAGGC | | 54.7 |
|  | Lht5R | TTGAAATCCGGCGGTCTATC | | 54.8 |
| PaLWMG0200007114.01 | Lht6F | TGGCTTGCCAAAGGATGCATTC | | 58.9 |
|  | Lht6R | TGTTGGAGCTAGAAGGTGTGGTC | | 59.1 |
| PaLWMG0400015688.01 | Lht7F | TAGACAAGGTCCGCGAAATGAC | | 57.2 |
|  | Lht7R | AACCGTGTCGTTCCCATATCC | | 57.3 |
| PaLWMG0400016836.01 | Lht8F | AAGAAGCCTATAACCGCCATCCC | | 59.4 |
|  | Lht8R | TGGAGCCGAGCTAACTCAGATTC | | 58.9 |
| PaLWMG0500019993.01 | Lht9F | TGGTGAACAAGGAGAAGGTGAG | | 56.8 |
|  | Lht9R | TTCTTGGTGGGAAGATTGGC | | 55.3 |
| PaLWMG0700026472.01 | Lht10F | ATGGCTGGCTTTCTTCTTCC | | 55.4 |
|  | Lht10R | AAATCCCATGTCCCATTGCC | | 55.8 |
| PaLWMG0600026219.01 | Hxjy1F | AAGGAAGGATGCACGCTCTG | | 58.0 |
|  | Hxjy1R | TTCAACTTGTCGGCCTTCTCC | | 57.7 |
| PaLWMG0100000820.01 | Hxjy2F | TTTCTAACCCTGGAGGAGCTGC | | 59.5 |
|  | Hxjy2R | CATTCACCAACACTGACTTGGG | | 56.4 |
| PaLWMG0300013271.01 | Hxjy3F | CCTGCTTGGAATTTGAGTTGGAG | | 56.4 |
|  | Hxjy3R | ACTTGGGAAGAGTCTGTGCATC | | 57.1 |
| PaLWMG0100001272.01 | Hxjy4F | ATGGGGTTTCAGTATGGGAGTG | | 56.8 |
|  | Hxjy4R | TCCACAGGCCTAGTTTCTGAAG | | 56.6 |
| PaLWMG0600024800.01 | Hxjy5F | GGGCAGTGTGCAAAAGGTTTC | | 57.8 |
|  | Hxjy5R | CTTGATCCCATTCCTCCATCGTG | | 57.9 |
| PaLWMG0400018716.01 | Hxjy6F | AGCCATCAGAGATTGGGTTCTC | | 56.8 |
|  | Hxjy6R | AGCAGCAGCACGTAAATTCC | | 56.0 |
| PaLWMG0400017153.01 | Hxjy7F | ACTCCTACCACAAACATGGG | | 54.5 |
|  | Hxjy7R | ACTGTCACACCAAGTTGACC | | 55.0 |

| Gene ID | Self-serial number | Primer sequence (5'-3') | Dissolving temperature（℃） | |
| --- | --- | --- | --- | --- |
| PaLWMG0100000305.01 | Hxjy8F | TGGCAGGAAGTGGTAGGATTAG | | 56.3 |
|  | Hxjy8R | AACCACACACGAATCCAACG | | 55.8 |
| PaLWMG0600023415.01 | Hxjy9F | GGCTATGCAAAGTGGGAATTCG | | 56.8 |
|  | Hxjy9R | AATGAACCCAGGGAAGCTTCTC | | 57.2 |
| PaLWMG0100000369.01 | Hxjy10F | ATCTCGCGCCACGGTAAATTG | | 58.4 |
|  | Hxjy10R | AGTTCCCGTTCTTGTCAAGCAG | | 57.7 |
| PaLWMG0100002125.01 | Sjb1F | CCCATTGAACTCTTCACCTAAGCTG | | 57.7 |
|  | Sjb1R | CTCTCAACAAATCCAGGGTCAGC | | 58.3 |
| PaLWMG0100002715.01 | Sjb2F | CACCAACAGCTAAGATGCAGG | | 56.4 |
|  | Sjb2R | AGACAGGAACAAGGCACAGAC | | 57.4 |
| PaLWMG0100002880.01 | Sjb3F | TTCCTCTTTCTCTCCACTTGGG | | 56.4 |
|  | Sjb3R | TGGTCACTTTCACAGTACAGAGAC | | 56.2 |
| PaLWMG0100003281.01 | Sjb4F | TTGATCCCTGTGCTAAGTGACC | | 56.9 |
|  | Sjb4R | CTGGCATTGAGGTTCACATTGG | | 56.8 |
| PaLWMG0100003413.01 | Sjb5F | TCGTCAACGAGTGCTCCTTC | | 57.4 |
|  | Sjb5R | CAAACTGCGCGAAACTCTGAG | | 57.1 |
| PaLWMG0100003852.01 | Sjb6F | TCAGCATCTGAACACCATCACC | | 57.4 |
|  | Sjb6R | CTTGGCGTCCATGATTTCTTGG | | 56.7 |
| PaLWMG0100004630.01 | Sjb7F | TTGCAGGCCATGGATCTCTTAG | | 57.1 |
|  | Sjb7R | TATTGCCGCACGATAGCCTATG | | 57.2 |
| PaLWMG0100005091.01 | Sjb8F | ACTCGCGACAGTCAATCCAG | | 57.5 |
|  | Sjb8R | AAGATAGCTCCACTGCCACTTC | | 56.9 |
| PaLWMG0100005997.01 | Sjb9F | TCACGGAATTGCTTCAGTCC | | 55.2 |
|  | Sjb9R | TGTTTGGTCCCTTTGACGTC | | 55.3 |
| PaLWMG0100006243.01 | Sjb10F | AATGAGTACCTTGCTGTCCTCAG | | 56.5 |
|  | Sjb10R | AGCCAAAGGTTGTTGCTTGC | | 57.1 |
| Actin | ActinF | ACATTGTTCTTAGTGGTGGGTC | | 54.6 |
|  | ActinR | AGATTCGTCATACTCTGCCTTT | | 53.1 |
| Cyclophilin | CyclophilinF | CAACGGATCTCAGTTCTTCGTCTGC | | 60.0 |
|  | CyclophilinR | GACCCAACCTTCTCGATGTTCTTCA | | 58.8 |

Table S2 Differential flavonoid substances in the fruits of two apricot species

| Material | Chemical formula | Material classification | | Material | Chemical formula | Material  classification |
| --- | --- | --- | --- | --- | --- | --- |
| Robinetin | C_15_H_10_O_7_ | Flavonols | | Isohyperoside | C_21_H_20_O_12_ | Flavonols |
| Quercetin | C_15_H_10_O_7_ | Flavonols | | Quercetin-5-O-glucuronide* | C_21_H_18_O_13_ | Flavonols |
| Tangeretin | C_20_H_20_O_7_ | Flavonols | | Quercetin-4'-O-glucuronide* | C_21_H_18_O_13_ | Flavonols |
| Kaempferol-3-O-arabinoside | C_20_H_18_O_10_ | Flavonols | | Herbacetin-3-O-glucuronide | C_21_H_18_O_13_ | Flavonols |
| Avicularin | C_20_H_18_O_11_ | Flavonols | | 6-Methoxykaempferol-3-O-glucoside | C_22_H_22_O_12_ | Flavonols |
| Quercetin-3-O-xyloside | C_20_H_18_O_11_ | Flavonols | | Rhamnetin-3-O-Glucoside | C_22_H_22_O_12_ | Flavonols |
| Kaempferol-7-O-glucoside | C_21_H_20_O_11_ | Flavonols | | Myricetin-3-O-glucoside* | C_21_H_20_O_13_ | Flavonols |
| Quercetin-3-O-rhamnoside | C_21_H_20_O_11_ | Flavonols | | Gossypetin-3-O-glucoside | C_21_H_20_O_13_ | Flavonols |
| Kaempferol-3-O-galactoside | C_21_H_20_O_11_ | Flavonols | | Myricetin-3-O-galactoside* | C_21_H_20_O_13_ | Flavonols |
| Kaempferol-4'-O-glucoside | C_21_H_20_O_11_ | Flavonols | | Kaempferol-3-O-(2''-acetyl)glucoside | C_23_H_22_O_12_ | Flavonols |
| Kaempferol-3-O-glucoside | C_21_H_20_O_11_ | Flavonols | | Kaempferol-3-O-(6''-acetyl)glucoside | C_23_H_22_O_12_ | Flavonols |
| Myricetin-3-O-arabinoside | C_20_H_18_O_12_ | Flavonols | | Kaempferol-3-O-(2''-O-acetyl)glucuronide | C_23_H_20_O_13_ | Flavonols |
| Kaempferol-3-O-glucuronide | C_21_H_18_O_12_ | Flavonols | | Quercetin-3-O-(6''-acetyl)glucoside | C_23_H_22_O_13_ | Flavonols |
| Azalein | C_22_H_22_O_11_ | Flavonols | | Quercetin-3-O-(6''-acetyl)galactoside | C_23_H_22_O_13_ | Flavonols |
| Material | Chemical formula | Material classification | | Material | Chemical formula | Material  classification |
| Rhamnetin-3-O-rhamnoside | C_22_H_22_O_11_ | Flavonols | | Isorhamnetin-3-O-(6''-acetylglucoside) | C_24_H_24_O_13_ | Flavonols |
| Quercetin-5-O-β-D-glucoside | C_21_H_20_O_12_ | Flavonols | | Kaempferol-3-O-(6''-malonyl)glucoside* | C_24_H_22_O_14_ | Flavonols |
| Quercetin-3-O-galactoside | C_21_H_20_O_12_ | Flavonols | | Kaempferol-3-O-(6''-malonyl)galactoside* | C_24_H_22_O_14_ | Flavonols |
| Quercetin-7-O-glucoside | C_21_H_20_O_12_ | Flavonols | | Quercetin-7-O-(6''-malonyl)glucoside | C_24_H_22_O_15_ | Flavonols |
| Quercetin-3-O-glucoside | C_21_H_20_O_12_ | Flavonols | | Kaempferol-3,7-O-dirhamnoside | C_27_H_30_O_14_ | Flavonols |
| Biondnoid I | C_30_H_26_O_13_ | Flavonols | | Kaempferol-3-O-(6''-p-Coumaroyl)glucoside | C_30_H_26_O_13_ | Flavonols |
| Kaempferol-3-O-glucorhamnoside | C_27_H_30_O_15_ | Flavonols | | Kaempferol-3-O-rutinoside | C_27_H_30_O_15_ | Flavonols |
| Kaempferol-3-O-rhamnosyl(1→2)glucoside | C_27_H_30_O_15_ | Flavonols | | Gossypetin-3-O-rutinoside | C_27_H_30_O_17_ | Flavonols |
| Kaempferol-3-O-neohesperidoside | C_27_H_30_O_15_ | Flavonols | Syringetin-3-O-rutinoside | | C_29_H_34_O_17_ | Flavonols |
| Quercetin-3-O-sambubioside | C_26_H_28_O_16_ | Flavonols | Kaempferol-3-O-robinoside-7-O-rhamnoside | | C_33_H_40_O_19_ | Flavonols |
| Quercetin-3-O-(6''-p-Coumaroyl)galactoside | C_30_H_26_O_14_ | Flavonols | quercetin 3-O-α-rhamnopyranosyl (1→2)-[α-rhamnopyranosyl (1→6)]-β-glucopyranoside | | C_33_H_40_O_20_ | Flavonols |
| Quercetin-3-O-robinobioside* | C_27_H_30_O_16_ | Flavonols | Quercetin-3-O-rutinoside-7-O-rhamnoside | | C_33_H_40_O_20_ | Flavonols |
| Quercetin-7-O-rutinoside* | C_27_H_30_O_16_ | Flavonols | Quercetin-3-O-(2''-O-Rhamnosyl)rutinoside | | C_33_H_40_O_20_ | Flavonols |
| Quercetin-3-O-rutinoside * | C_27_H_30_O_16_ | Flavonols | Quercetin-3-O-sophoroside-7-O-rhamnoside | | C_33_H_40_O_21_ | Flavonols |
| Quercetin-3-O-neohesperidoside* | C_27_H_30_O_16_ | Flavonols | 6-Hydroxykaempferol 3-Rutinoside-6-glucoside | | C_33_H_40_O_21_ | Flavonols |
| Rhamnetin-3-O-Rutinoside | C_28_H_32_O_16_ | Flavonols | Quercetin-3-O-rutinoside-7-O-glucoside | | C_33_H_40_O_21_ | Flavonols |
| Tamarixetin-3-O-rutinoside | C_28_H_32_O_16_ | Flavonols | Apigenin-7-O-glucuronide | | C_21_H_18_O_11_ | Flavones |
| Tamarixetin-3-O-glucoside-7-O-rhamnoside | C_28_H_32_O_16_ | Flavonols | 7,4'-Dihydroxyflavone | | C_15_H_10_O_4_ | Flavones |
| Isorhamnetin-3-O-rutinoside | C_28_H_32_O_16_ | Flavonols | Baicalein | | C_15_H_10_O_5_ | Flavones |
| Sexangularetin-3-O-glucoside-7-O-rhamnoside | C_28_H_32_O_16_ | Flavonols | Apiferol | | C_15_H_14_O_5_ | Flavones |
| Isorhamnetin-3-O-neohespeidoside | C_28_H_32_O_16_ | Flavonols | Acacetin | | C_16_H_12_O_5_ | Flavones |
| Myricetin-3-O-galactoside-3'-O-rhamnoside | C_27_H_30_O_17_ | Flavonols | Genkwanin | | C_16_H_12_O_5_ | Flavones |
| Quercetin-5,4'-di-O-glucoside | C_27_H_30_O_17_ | Flavonols | Wogonin | | C_16_H_12_O_5_ | Flavones |
| Quercetin-3-O-sophoroside | C_27_H_30_O_17_ | Flavonols | Mearnsetin | | C_16_H_12_O_8_ | Flavones |
| 6-Hydroxykaempferol 6,7-Diglucoside | C_27_H_30_O_17_ | Flavonols | 5-Hydroxy-6,7,3',4'-tetramethoxyflavone | | C_19_H_18_O_7_ | Flavones |
| 6-Hydroxykaempferol 3,6-Diglucoside | C_27_H_30_O_17_ | Flavonols | Pinocembrin-7-O-glucoside | | C_21_H_22_O_9_ | Flavones |
| 6-Hydroxykaempferol-3,6-O-Diglucoside | C_27_H_30_O_17_ | Flavonols | Baicalin | | C_21_H_18_O_11_ | Flavones |
| Myricetin-3-O-rutinoside | C_27_H_30_O_17_ | Flavonols | Luteolin-7-O-glucoside | | C_21_H_20_O_11_ | Flavones |
| Quercetin-7-O-rutinoside-4'-O-glucoside | C_33_H_40_O_21_ | Flavonols | Luteolin-3'-O-glucoside | | C_21_H_20_O_11_ | Flavones |
| Wogonin-7-O-Glucuronide | C_22_H_20_O_11_ | Flavones | Luteolin-7-O-neohesperidoside | | C_27_H_30_O_15_ | Flavones |
| Material | Chemical formula | Material classification | Material | | Chemical formula | Material  classification |
| Oroxylin A-7-O-glucuronide | C_22_H_20_O_11_ | Flavones | Tricin-7-O-(2''-feruloyl)glucoside | | C_33_H_32_O_15_ | Flavones |
| Acacetin-7-O-glucuronide | C_26_H_20_O_8_ | Flavones | Pelargonidin-3-O-glucoside | | C_21_H_21_O_10_+ | Anthocyanidins |
| Luteolin-7-O-glucuronide | C_21_H_18_O_12_ | Flavones | Delphinidin-3-O-arabinoside | | C_20_H_19_O_11_+ | Anthocyanidins |
| Tetahydroxyflavone-7-O-glucuronide | C_21_H_18_O_12_ | Flavones | Petunidin-3-O-arabinoside | | C_21_H_21_O_11_+ | Anthocyanidins |
| Scutellarein-7-O-glucuronide | C_21_H_18_O_12_ | Flavones | Delphinidin-3-O-glucoside | | C_21_H_21_O_12_+ | Anthocyanidins |
| Yuanhuanin | C_22_H_22_O_11_ | Flavones | Delphinidin-3-O-glucuronide | | C_21_H_19_O_13_+ | Anthocyanidins |
| Chrysoeriol-6-C-glucoside | C_22_H_22_O_11_ | Flavones | Malvidin-3-O-glucoside | | C_23_H_25_O_12_+ | Anthocyanidins |
| Chrysoeriol-5-O-glucoside | C_22_H_22_O_11_ | Flavones | Pelargonidin-3-O-rutinoside | | C_27_H_31_O_14_+ | Anthocyanidins |
| 6-Hydroxyluteolin 5-glucoside | C_21_H_20_O_12_ | Flavones | Cyanidin-3-O-sambubioside [Cyanidin-3-O-(2''-O-xylosyl)glucoside] | | C_26_H_29_O_15_+ | Anthocyanidins |
| Nepetin-7-O-glucoside | C_22_H_22_O_12_ | Flavones | Cyanidin-3-O-(6''-O-p-Coumaroyl)glucoside | | C_30_H_27_O_13_+ | Anthocyanidins |
| Nepetin-7-O-alloside | C_22_H_22_O_12_ | Flavones | Cyanidin-3-O-(6''-O-caffeoyl)glucoside | | C_30_H_27_O_14_+ | Anthocyanidins |
| 3',5',5,7-Tetrahydroxy-4'-methoxyflavanone-3'-O-glucoside | C_22_H_24_O_12_ | Flavones | Delphinidin-3-O-(6''-O-p-coumaroyl)glucoside | | C_30_H_27_O_14_+ | Anthocyanidins |
| Tricin-5-O-Glucoside | C_23_H_24_O_12_ | Flavones | Petunidin-3-O-(6''-O-p-Coumaroyl)glucoside | | C_31_H_29_O_14_+ | Anthocyanidins |
| Mearnsetin-3-O-glucoside | C_22_H_22_O_13_ | Flavones | Delphinidin-3-O-(6''-O-caffeoyl)glucoside | | C_30_H_27_O_15_+ | Anthocyanidins |
| Tricin-7-O-Glucuronide | C_23_H_22_O_13_ | Flavones | Delphinidin-3,5-di-O-glucoside | | C_27_H_31_O_17_+ | Anthocyanidins |
| Material | Chemical formula | Material classification | Material | | Chemical formula | Material  classification |
| Tricin-7-O-saccharic acid | C_23_H_22_O_14_ | Flavones | Cyanidin-3-O-(6''-O-caffeoyl-2''-O-xylosyl)glucoside | | C_35_H_35_O_18_+ | Anthocyanidins |
| Isorhamnetin-3-O-(6''-malonylglucoside) | C_25_H_24_O_15_ | Flavones | Delphinidin-3-O-(2'''-O-p-coumaroyl)rutinoside | | C_33_H_41_O_20_+ | Anthocyanidins |
| Apigenin-7-O-rutinoside | C_27_H_30_O_14_ | Flavones | Petunidin-3-O-(6''-O-p-coumaroyl)glucoside-5-O-rhamnoside | | C_37_H_39_O_18_+ | Anthocyanidins |
| Luteolin-7-O-(6''-caffeoyl)rhamnoside | C_30_H_26_O_13_ | Flavones | Delphinidin-3-O-rutinoside-7-O-glucoside | | C_33_H_41_O_21_+ | Anthocyanidins |
| Kaempferol-3-O-(6''-p-Coumaroyl)galactoside | C_30_H_26_O_13_ | Flavones | Cyanidin-3-O-sophoroside-5-O-glucoside | | C_33_H_41_O_21_+ | Anthocyanidins |
| Luteolin-7-O-rutinoside | C_27_H_30_O_15_ | Flavones | Delphinidin-3-O-(2'''-O-p-coumaroyl)rutinoside-5-O-glucoside | | C_42_H_47_O_23_+ | Anthocyanidins |
| Gallic acid | C_7_H_6_O_5_ | Tannin | Epitheaflavic acid-3-O-Gallate | | C_28_H_20_O_14_ | Flavanols |
| 1-O-Galloyl-D-glucose* | C_13_H_16_O_10_ | Tannin | Catechin-catechin-catechin | | C_45_H_38_O_18_ | Flavanols |
| 6-O-Galloyl-glucose* | C_13_H_16_O_10_ | Tannin | Epicatechin glucoside | | C_21_H_24_O_11_ | Flavanols |
| 3-O-Galloyl-glucose* | C_13_H_16_O_10_ | Tannin | Epicatechin-3'-O-β-D-glucopyranoside | | C_21_H_24_O_11_ | Flavanols |
| p-Dimeric galloyl methyl ester | C_15_H_12_O_9_ | Tannin | Epicatechin-4'-O-β-D-glucopyranoside | | C_21_H_24_O_11_ | Flavanols |
| Galloyl Methyl gallate | C_15_H_12_O_9_ | Tannin | Epigallocatechin-3-gallate* | | C_22_H_18_O_11_ | Flavanols |
| 2-O-Salicyl-6-O-Galloyl-D-Glucose | C_20_H_20_O_12_ | Tannin | Gallate catechin gallate* | | C_22_H_18_O_11_ | Flavanols |
| (-)-Epicatechin-3-(3''-O-methyl)gallate | C_23_H_20_O_10_ | Tannin | Gallocatechin 3-O-gallate | | C_22_H_18_O_11_ | Flavanols |
| Material | Chemical formula | Material classification | Material | | Chemical formula | Material  classification |
| 3,4-Digalloylshikimic acid | C_21_H_18_O_13_ | Tannin | Epicatechin-epiafzelechin | | C_30_H_26_O_11_ | Flavanols |
| 3-O-Digalloyl quinic acid | C_21_H_20_O_14_ | Tannin | Tetrahydroxyflavan-(4α-8-epicatechin) | | C_30_H_24_O_12_ | Flavanols |
| Gambiriin B3 | C_30_H_26_O_11_ | Tannin | 6-C-Glucosyl-2-Hydroxynaringenin | | C_21_H_22_O_11_ | Flavanones |
| Sanguiin H4 | C_27_H_22_O_18_ | Tannin | Eriodictyol-3'-O-glucoside | | C_21_H_22_O_11_ | Flavanones |
| Galloyl-ellagyl-glucose | C_27_H_22_O_18_ | Tannin | Naringenin (5,7,4'-Trihydroxyflavanone) | | C_15_H_12_O_5_ | Flavanones |
| Sanguiin H1 | C_34_H_26_O_22_ | Tannin | Eriodictyol (5,7,3',4'-Tetrahydroxyflavanone) | | C_15_H_12_O_6_ | Flavanones |
| Arecatannin C1 | C_45_H_38_O_18_ | Tannin | Hesperetin | | C_16_H_14_O_6_ | Flavanones |
| Arecatannin B1 | C_45_H_38_O_18_ | Tannin | Butin-7-O-glucoside | | C_21_H_22_O_10_ | Flavanones |
| Epicatechin | C_15_H_14_O_6_ | Flavanols | Naringenin-7-O-glucoside (Prunin) | | C_21_H_22_O_10_ | Flavanones |
| 5,7,3',4',5'-Pentahydroxyflavan (Tricetiflavan) | C_15_H_14_O_6_ | Flavanols | Naringenin-4'-O-glucoside | | C_21_H_22_O_10_ | Flavanones |
| Epigallocatechin | C_15_H_14_O_7_ | Flavanols | eriodictyol 7-O-β-D-glucopyranoside | | C_21_H_22_O_11_ | Flavanones |
| Gallocatechin | C_15_H_14_O_7_ | Flavanols | 3',5,5',7-Tetrahydroxyflavanone-7-O-glucoside | | C_21_H_22_O_11_ | Flavanones |
| 7-O-Galloyltricetiflavan | C_22_H_18_O_10_ | Flavanols | Eriodictyol-7-O-glucoside | | C_21_H_22_O_11_ | Flavanones |
| Catechin gallate | C_22_H_18_O_10_ | Flavanols | Eriodictyol-8-C-glucoside | | C_21_H_22_O_11_ | Flavanones |
| Catechin-(7,8-bc)-4α-(3,4-dihydroxyphenyl)-dihydro-2-(3H)-one | C_24_H_20_O_9_ | Flavanols | Persicoside | | C_23_H_26_O_11_ | Flavanones |
| Catechin-(7,8-bc)-4β-(3,4-dihydroxyphenyl)-dihydro-2-(3H)-one | C_24_H_20_O_9_ | Flavanols | Hesperetin-7-O-rutinoside (Hesperidin) | | C_28_H_34_O_15_ | Flavanones |
| Material | Chemical formula | Material classification | Material | | Chemical formula | Material  classification |
| Hesperetin-7-O-neohesperidoside | C_28_H_34_O_15_ | Flavanones | Phellamurin | | C_26_H_30_O_11_ | Flavanonols |
| Butein | C_15_H_12_O_5_ | Chalcones | 2α,3α-Epoxy-5,7,3',4'-tetrahydroxyflavan-(4β-8-catechin) | | C_30_H_24_O_12_ | Proanthocyanidins |
| Naringenin chalcone | C_15_H_12_O_5_ | Chalcones | 2α,3α-Epoxy-5,7,3',4'-tetrahydroxyflavan-(4β-8-epicatechin) | | C_30_H_24_O_12_ | Proanthocyanidins |
| 2,4,2',4'-tetrahydroxy-3'-prenylchalcone | C_20_H_20_O_5_ | Chalcones | Procyanidin A2 | | C_30_H_24_O_12_ | Proanthocyanidins |
| Isosalipurposide (Phlorizin Chalcone) | C_21_H_22_O_10_ | Chalcones | Procyanidin B3 | | C_30_H_26_O_12_ | Proanthocyanidins |
| Dihydrocharcone-4'-O-glucoside | C_21_H_24_O_10_ | Chalcones | Procyanidin B2 | | C_30_H_26_O_12_ | Proanthocyanidins |
| Phloretin-2'-O-glucoside (Phlorizin) | C_21_H_24_O_10_ | Chalcones | Procyanidin B1 | | C_30_H_26_O_12_ | Proanthocyanidins |
| Carthamone | C_21_H_20_O_11_ | Chalcones | Procyanidin B4 | | C_30_H_26_O_12_ | Proanthocyanidins |
| Okanin-4'-O-glucoside(Marein) | C_21_H_22_O_11_ | Chalcones | Galloylprocyanidin B4 | | C_37_H_30_O_16_ | Proanthocyanidins |
| 3,4,2',4',6'-Pentahydroxychalcone-4'-O-glucoside | C_21_H_22_O_11_ | Chalcones | Procyanidin C2 | | C_45_H_38_O_18_ | Proanthocyanidins |
| Sieboldin | C_21_H_24_O_11_ | Chalcones | Procyanidin C1 | | C_45_H_38_O_18_ | Proanthocyanidins |
| Phloretin-4'-O-(6''-Caffeoyl)glucoside | C_30_H_30_O_13_ | Chalcones | Apigenin-6,8-di-C-glucoside (Vicenin-2) | | C_27_H_30_O_15_ | Flavonoid carbonoside |
| Pinobanksin | C_15_H_12_O_5_ | Flavanonols | Orientin-7-O-glucoside | | C_27_H_30_O_16_ | Flavonoid carbonoside |
| Dihydrokaempferide | C_16_H_14_O_6_ | Flavanonols | Hesperetin-8-C-glucoside-3'-O-glucoside | | C_28_H_34_O_16_ | Flavonoid carbonoside |
| Dihydroquercetin(Taxifolin) | C_15_H_12_O_7_ | Flavanonols | Apigenin-8-C-glucoside-7-O-Sophoroside | | C_33_H_40_O_20_ | Flavonoid carbonoside |
| Taxifolin-3-O-rhamnoside (Astilbin) | C_21_H_22_O_11_ | Flavanonols | Apigenin-6-C-(2''-glucuronyl)xyloside | | C_26_H_26_O_15_ | Flavonoid carbonoside |
| Dihydrokaempferol-3-O-glucoside | C_21_H_22_O_11_ | Flavanonols | Biochanin A | | C_16_H_12_O_5_ | Isoflavones |
| Dihydrokaempferol-7-O-glucoside | C_21_H_22_O_11_ | Flavanonols | Genistein-7-O-galactoside-rhamnose | | C_27_H_30_O_14_ | Isoflavones |
| Aromadendrin-7-O-glucoside | C_21_H_22_O_11_ | Flavanonols | 2'-Hydoxy,5-methoxyGenistein-O-rhamnosyl-glucoside | | C_28_H_32_O_16_ | Isoflavones |
| Hesperetin-5-O-glucoside | C_22_H_24_O_11_ | Flavanonols | 3,9-Dihydroxypterocarpan | | C_15_H_12_O_4_ | Isoflavones |
| Taxifolin-3'-O-glucoside | C_21_H_22_O_12_ | Flavanonols |  | |  |  |

Table S3 Summary of sample sequencing data quality

| Sample number | Raw reads | Clean reads | Clean base(G) | Error rate(%) | Q20 (%) | Q30 (%) | GC content(%) |
| --- | --- | --- | --- | --- | --- | --- | --- |
| ZSS1-1 | 52498004 | 50136894 | 7.52 | 0.03 | 97.58 | 93.05 | 45.78 |
| ZSS1-2 | 43270808 | 41656454 | 6.25 | 0.03 | 97.65 | 93.19 | 46.39 |
| ZSS1-3 | 47129300 | 45427268 | 6.81 | 0.03 | 97.63 | 93.1 | 46.51 |
| ZSS2-1 | 43293536 | 41713012 | 6.26 | 0.03 | 97.26 | 92.43 | 45.89 |
| ZSS2-2 | 48048942 | 45814762 | 6.87 | 0.03 | 97.51 | 92.89 | 45.93 |
| ZSS2-3 | 47877832 | 45500988 | 6.83 | 0.03 | 97.63 | 93.16 | 45.89 |
| ZSS3-1 | 48581124 | 45605272 | 6.84 | 0.03 | 97.47 | 92.88 | 45.46 |
| ZSS3-2 | 45319522 | 43020988 | 6.45 | 0.03 | 97.73 | 93.37 | 46.03 |
| ZSS3-3 | 45808674 | 43334932 | 6.5 | 0.03 | 97.4 | 92.64 | 46.03 |
| ZSS4-1 | 45657838 | 43641562 | 6.55 | 0.03 | 97.65 | 93.13 | 45.82 |
| ZSS4-2 | 47747902 | 43489324 | 6.52 | 0.03 | 97.88 | 93.69 | 45.8 |
| ZSS4-3 | 44893080 | 40673760 | 6.1 | 0.03 | 98.07 | 94.19 | 45.6 |
| ZSS5-1 | 45803472 | 43623876 | 6.54 | 0.03 | 97.8 | 93.51 | 45.79 |
| ZSS5-2 | 47621994 | 46015912 | 6.9 | 0.03 | 97.66 | 93.14 | 45.81 |
| ZSS5-3 | 45665306 | 43897004 | 6.58 | 0.03 | 97.78 | 93.43 | 45.91 |
| JNS1-1 | 51557190 | 47355332 | 7.1 | 0.03 | 98.01 | 94.02 | 44.81 |
| JNS1-2 | 53715524 | 49551784 | 7.43 | 0.03 | 97.68 | 93.27 | 46.25 |
| JNS1-3 | 61327158 | 57231530 | 8.58 | 0.03 | 97.66 | 93.2 | 46.18 |
| JNS2-1 | 53979658 | 50557996 | 7.58 | 0.03 | 97.63 | 93.11 | 45.14 |
| JNS2-2 | 56897666 | 54959806 | 8.24 | 0.03 | 97.48 | 92.82 | 46.04 |
| JNS2-3 | 49843538 | 48283152 | 7.24 | 0.03 | 97.57 | 93.07 | 46.05 |
| JNS3-1 | 48212880 | 46484458 | 6.97 | 0.03 | 97.46 | 92.9 | 45.74 |
| JNS3-2 | 52222614 | 50538052 | 7.58 | 0.03 | 97.44 | 92.74 | 45.71 |
| JNS3-3 | 47877694 | 46174730 | 6.93 | 0.03 | 97.6 | 93.11 | 45.79 |
| JNS4-1 | 45854102 | 44011096 | 6.6 | 0.03 | 97.64 | 93.13 | 45.66 |
| JNS4-2 | 47655558 | 44754642 | 6.71 | 0.03 | 97.74 | 93.35 | 45.15 |
| JNS4-3 | 45618700 | 43332434 | 6.5 | 0.03 | 97.94 | 93.84 | 45.74 |
| JNS5-1 | 43156846 | 40761642 | 6.11 | 0.03 | 97.71 | 93.28 | 45.78 |
| JNS5-2 | 59063146 | 55687648 | 8.35 | 0.03 | 97.67 | 93.19 | 45.76 |
| JNS5-3 | 51992328 | 48716426 | 7.31 | 0.03 | 97.74 | 93.35 | 45.7 |

Table S4 Transcriptome data and reference genome alignment results

| Sample number | Total reads | Mapped reads | Mapped rate（%） |
| --- | --- | --- | --- |
| ZSS1-1 | 50136894 | 47327734 | 0.944 |
| ZSS1-2 | 41656454 | 39250634 | 0.9422 |
| ZSS1-3 | 45427268 | 42876418 | 0.9438 |
| ZSS2-1 | 41713012 | 38824927 | 0.9308 |
| ZSS2-2 | 45814762 | 42939224 | 0.9372 |
| ZSS2-3 | 45500988 | 42680495 | 0.938 |
| ZSS3-1 | 45605272 | 42455640 | 0.9309 |
| ZSS3-2 | 43020988 | 40320188 | 0.9372 |
| ZSS3-3 | 43334932 | 40526775 | 0.9352 |
| ZSS4-1 | 43641562 | 41168659 | 0.9433 |
| ZSS4-2 | 43489324 | 40953584 | 0.9417 |
| ZSS4-3 | 40673760 | 38401338 | 0.9441 |
| ZSS5-1 | 43623876 | 41191877 | 0.9443 |
| ZSS5-2 | 46015912 | 43454602 | 0.9443 |
| ZSS5-3 | 43897004 | 41558741 | 0.9467 |
| JNS1-1 | 47355332 | 44570696 | 0.9412 |
| JNS1-2 | 49551784 | 46630985 | 0.9411 |
| JNS1-3 | 57231530 | 53964091 | 0.9429 |
| JNS2-1 | 50557996 | 47529330 | 0.9401 |
| JNS2-2 | 54959806 | 51416795 | 0.9355 |
| JNS2-3 | 48283152 | 45225831 | 0.9367 |
| JNS3-1 | 46484458 | 43493048 | 0.9356 |
| JNS3-2 | 50538052 | 47375254 | 0.9374 |
| JNS3-3 | 46174730 | 43410811 | 0.9401 |
| JNS4-1 | 44011096 | 41629843 | 0.9459 |
| JNS4-2 | 44754642 | 42344577 | 0.9461 |
| JNS4-3 | 43332434 | 41122937 | 0.949 |
| JNS5-1 | 40761642 | 38683808 | 0.949 |
| JNS5-2 | 55687648 | 52779752 | 0.9478 |
| JNS5-3 | 48716426 | 46184687 | 0.948 |

Table S5 Core genes in the flavonoid biosynthesis pathway of two apricot species

|  |  | FPKM | | | | | | | | | |
| --- | --- | --- | --- | --- | --- | --- | --- | --- | --- | --- | --- |
| ID | Self-numbering | ZSS1 | ZSS2 | ZSS3 | ZSS4 | ZSS5 | JNS1 | JNS2 | JNS3 | JNS4 | JNS5 |
| PaLWMG0600022327.01 | C4H | 114.11 | 59.42 | 63.24 | 5.75 | 7.68 | 70.92 | 54.17 | 37.99 | 6.38 | 0.89 |
| PaLWMG0100000032.01 | CHS1 | 57.68 | 3.15 | 8.70 | 0.14 | 0.10 | 14.33 | 2.89 | 13.10 | 0.33 | 0.09 |
| PaLWMG0100000037.01 | CHS2 | 70.65 | 7.77 | 12.72 | 0.46 | 0.92 | 26.69 | 8.55 | 47.22 | 5.95 | 1.25 |
| PaLWMG0200009862.01 | CHI1 | 75.97 | 42.15 | 74.70 | 28.22 | 43.27 | 35.95 | 32.41 | 59.43 | 41.38 | 26.13 |
| PaLWMG0200010310.01 | CHI2 | 116.47 | 25.11 | 126.26 | 82.08 | 140.19 | 48.58 | 28.60 | 85.02 | 181.63 | 93.30 |
| PaLWMG0400016860.01 | CHI3 | 9.46 | 5.01 | 3.96 | 6.62 | 5.83 | 13.86 | 8.02 | 3.78 | 9.96 | 5.93 |
| PaLWMG0100003854.01 | LAR | 75.77 | 5.71 | 7.94 | 2.88 | 1.46 | 36.15 | 5.76 | 13.70 | 9.86 | 0.99 |
| PaLWMG0100004646.01 | DFR1 | 98.25 | 10.31 | 25.55 | 9.60 | 3.61 | 43.12 | 7.66 | 27.73 | 12.98 | 0.40 |
| PaLWMG0200008916.01 | DFR2 | 7.93 | 13.01 | 13.51 | 12.96 | 9.14 | 9.65 | 31.32 | 83.06 | 63.22 | 41.24 |
| PaLWMG0300014662.01 | DFR3 | 19.85 | 10.79 | 9.18 | 16.41 | 14.94 | 16.55 | 12.47 | 7.73 | 21.26 | 14.29 |
| PaLWMG0400016256.01 | DFR4 | 0.03 | 0.92 | 7.02 | 45.78 | 49.39 | 0.03 | 0.34 | 28.84 | 79.64 | 51.95 |
| PaLWMG0100005999.01 | FLS1 | 54.05 | 8.39 | 14.99 | 0.00 | 0.03 | 0.54 | 0.45 | 0.39 | 0.07 | 0.00 |
| PaLWMG0200007112.01 | FLS2 | 17.16 | 14.35 | 18.63 | 33.67 | 43.25 | 24.63 | 22.13 | 23.99 | 74.20 | 54.22 |
| PaLWMG0200007114.01 | FLS3 | 9.62 | 13.34 | 16.87 | 24.08 | 25.24 | 10.08 | 15.96 | 10.90 | 30.55 | 20.08 |
| PaLWMG0200009277.01 | UGT79B11 | 58.90 | 5.19 | 3.84 | 0.92 | 1.83 | 7.86 | 3.24 | 3.53 | 1.70 | 0.98 |
| PaLWMG0200009279.01 | UGT79B12 | 28.48 | 13.95 | 17.32 | 50.46 | 35.83 | 0.15 | 0.21 | 0.10 | 0.17 | 0.05 |
| PaLWMG0400016590.01 | UGT79B13 | 39.51 | 40.36 | 33.20 | 21.55 | 11.29 | 142.92 | 128.73 | 62.45 | 133.25 | 78.46 |
| PaLWMG0600024364.01 | UGT79B14 | 112.45 | 101.94 | 147.30 | 96.16 | 22.93 | 99.43 | 84.19 | 74.43 | 104.90 | 32.15 |
| PaLWMG0600024371.01 | UGT79B15 | 0.07 | 0.30 | 1.47 | 7.94 | 8.69 | 0.30 | 2.04 | 4.40 | 10.24 | 11.23 |
| PaLWMG0700027025.01 | UGT79B16 | 43.52 | 84.01 | 92.33 | 86.96 | 9.71 | 28.71 | 84.15 | 65.79 | 46.89 | 13.66 |
| PaLWMG0300012746.01 | ANS1 | 8.56 | 7.40 | 10.06 | 11.14 | 18.73 | 7.51 | 8.14 | 9.82 | 17.53 | 16.46 |
| PaLWMG0400015680.01 | ANS2 | 3.75 | 5.52 | 7.29 | 10.25 | 8.55 | 7.45 | 10.66 | 10.86 | 15.36 | 12.99 |
| PaLWMG0400015684.01 | ANS3 | 41.30 | 149.34 | 97.25 | 35.06 | 8.24 | 22.83 | 41.58 | 12.77 | 2.55 | 0.72 |
| PaLWMG0400015688.01 | ANS4 | 13.28 | 30.74 | 39.19 | 42.05 | 19.87 | 31.31 | 46.89 | 51.10 | 23.03 | 14.11 |
| PaLWMG0400015691.01 | ANS5 | 1.74 | 4.70 | 5.29 | 4.25 | 1.50 | 8.08 | 12.34 | 11.60 | 4.35 | 1.97 |
| PaLWMG0400016836.01 | ANS6 | 7.95 | 14.85 | 16.20 | 17.48 | 16.81 | 0.92 | 1.19 | 5.57 | 9.53 | 5.30 |
| PaLWMG0500019694.01 | ANS7 | 9.11 | 18.45 | 21.08 | 7.21 | 1.53 | 10.52 | 16.64 | 19.50 | 5.25 | 1.38 |
| PaLWMG0500019993.01 | ANS8 | 111.68 | 11.39 | 12.06 | 9.37 | 7.64 | 67.27 | 15.34 | 23.17 | 38.36 | 12.48 |
| PaLWMG0700026472.01 | ANS9 | 46.10 | 80.16 | 110.57 | 272.81 | 190.53 | 55.07 | 88.65 | 119.27 | 336.04 | 300.66 |
| PaLWMG0400018467.01 | ANR1 | 47.67 | 15.46 | 20.37 | 11.44 | 8.50 | 25.06 | 17.86 | 32.63 | 11.28 | 7.13 |
| PaLWMG0500020310.01 | ANR2 | 14.32 | 35.87 | 32.43 | 17.40 | 9.09 | 13.63 | 36.05 | 42.02 | 24.66 | 7.89 |
| PaLWMG0700027450.01 | F3H | 43.48 | 41.99 | 76.89 | 93.16 | 38.82 | 33.13 | 52.58 | 205.64 | 37.42 | 14.21 |
| PaLWMG0500019860.01 | CYP75B11 | 10.35 | 15.01 | 14.19 | 5.48 | 1.32 | 8.51 | 14.24 | 17.96 | 5.36 | 1.23 |
| PaLWMG0500021350.01 | CYP75B12 | 351.87 | 262.89 | 367.49 | 114.06 | 140.18 | 176.08 | 225.08 | 297.45 | 362.10 | 50.31 |

Table S6 Transcription factors of different modules in WGCNA analysis

| MYB | Gene ID | TFs |
| --- | --- | --- |
|  | PaLWMG0100004067.01 | MYB1 |
|  | PaLWMG0100004950.01 | MYB2 |
|  | PaLWMG0100005213.01 | MYB3 |
|  | PaLWMG0100005336.01 | MYB4 |
|  | PaLWMG0200009477.01 | MYB5 |
|  | PaLWMG0200010099.01 | MYB6 |
|  | PaLWMG0300013086.01 | MYB7 |
|  | PaLWMG0300014515.01 | MYB8 |
|  | PaLWMG0400018417.01 | MYB9 |
|  | PaLWMG0500021515.01 | MYB10 |
|  | PaLWMG0500021570.01 | MYB11 |
|  | PaLWMG0600024647.01 | MYB12 |
|  | PaLWMG0600024954.01 | MYB13 |
|  | PaLWMG0600024988.01 | MYB14 |
|  | PaLWMG0600025150.01 | MYB15 |
|  | PaLWMG0700026541.01 | MYB16 |
|  | PaLWMG0800030198.01 | MYB17 |
|  | PaLWMG0800031281.01 | MYB18 |
| bHLH | Gene ID | TFs |
|  | PaLWMG0100003489.01 | bHLH1 |
|  | PaLWMG0100003767.01 | bHLH2 |
|  | PaLWMG0100005091.01 | bHLH3 |
|  | PaLWMG0100006243.01 | bHLH4 |
|  | PaLWMG0200007124.01 | bHLH5 |
|  | PaLWMG0200009510.01 | bHLH6 |
|  | PaLWMG0300011657.01 | bHLH7 |
|  | PaLWMG0300012807.01 | bHLH8 |
|  | PaLWMG0400018068.01 | bHLH9 |
|  | PaLWMG0500020149.01 | bHLH10 |
|  | PaLWMG0500021147.01 | bHLH11 |
|  | PaLWMG0500021419.01 | bHLH12 |
|  | PaLWMG0600022102.01 | bHLH13 |
|  | PaLWMG0600024432.01 | bHLH14 |
|  | PaLWMG0600025999.01 | bHLH15 |
|  | PaLWMG0700027864.01 | bHLH16 |
|  | PaLWMG0700028578.01 | bHLH17 |
|  | PaLWMG0800029973.01 | bHLH18 |
|  | PaLWMG0800030071.01 | bHLH19 |
|  | PaLWMG0800031025.01 | bHLH20 |
|  | PaLWMG0800031715.01 | bHLH21 |


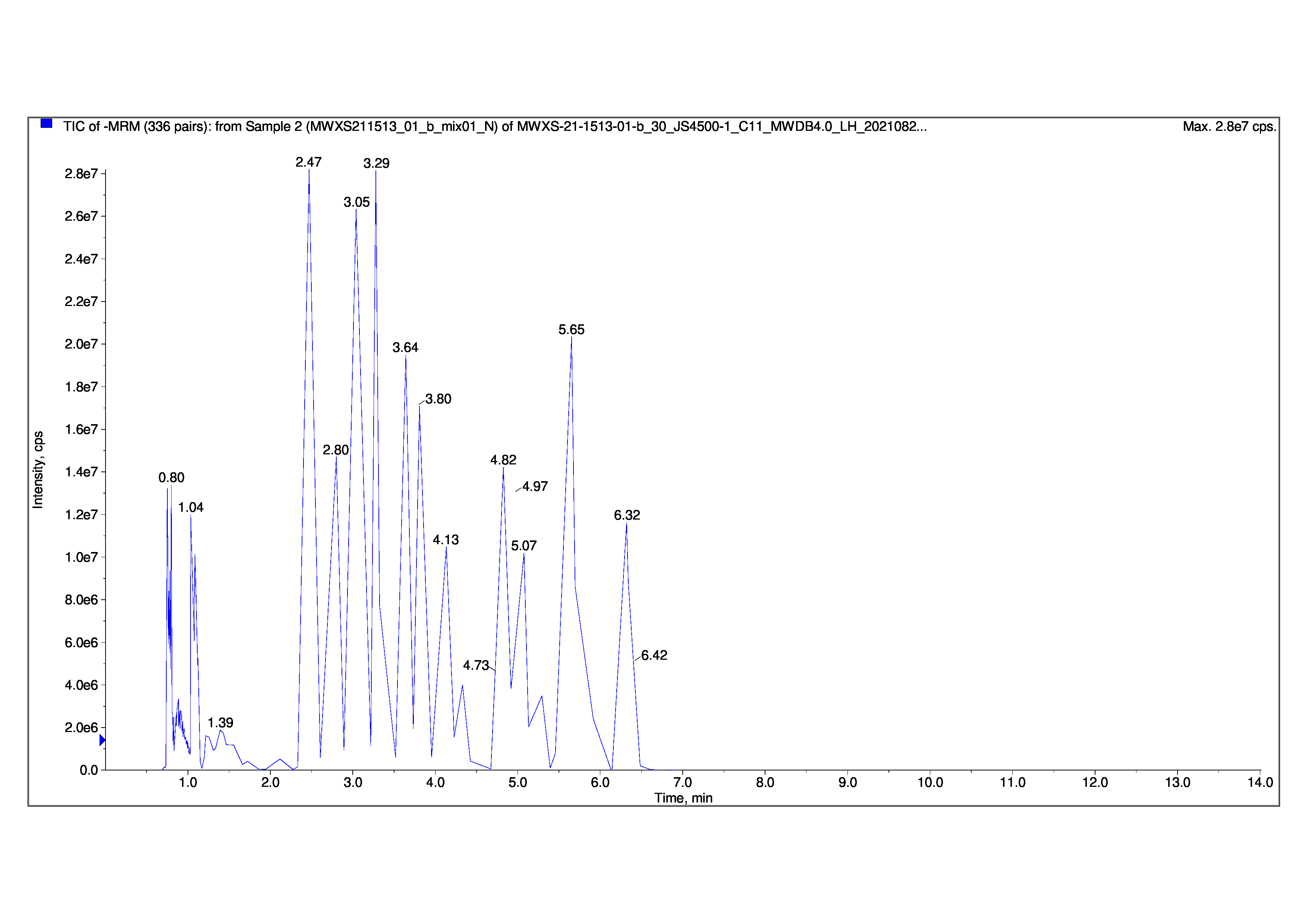

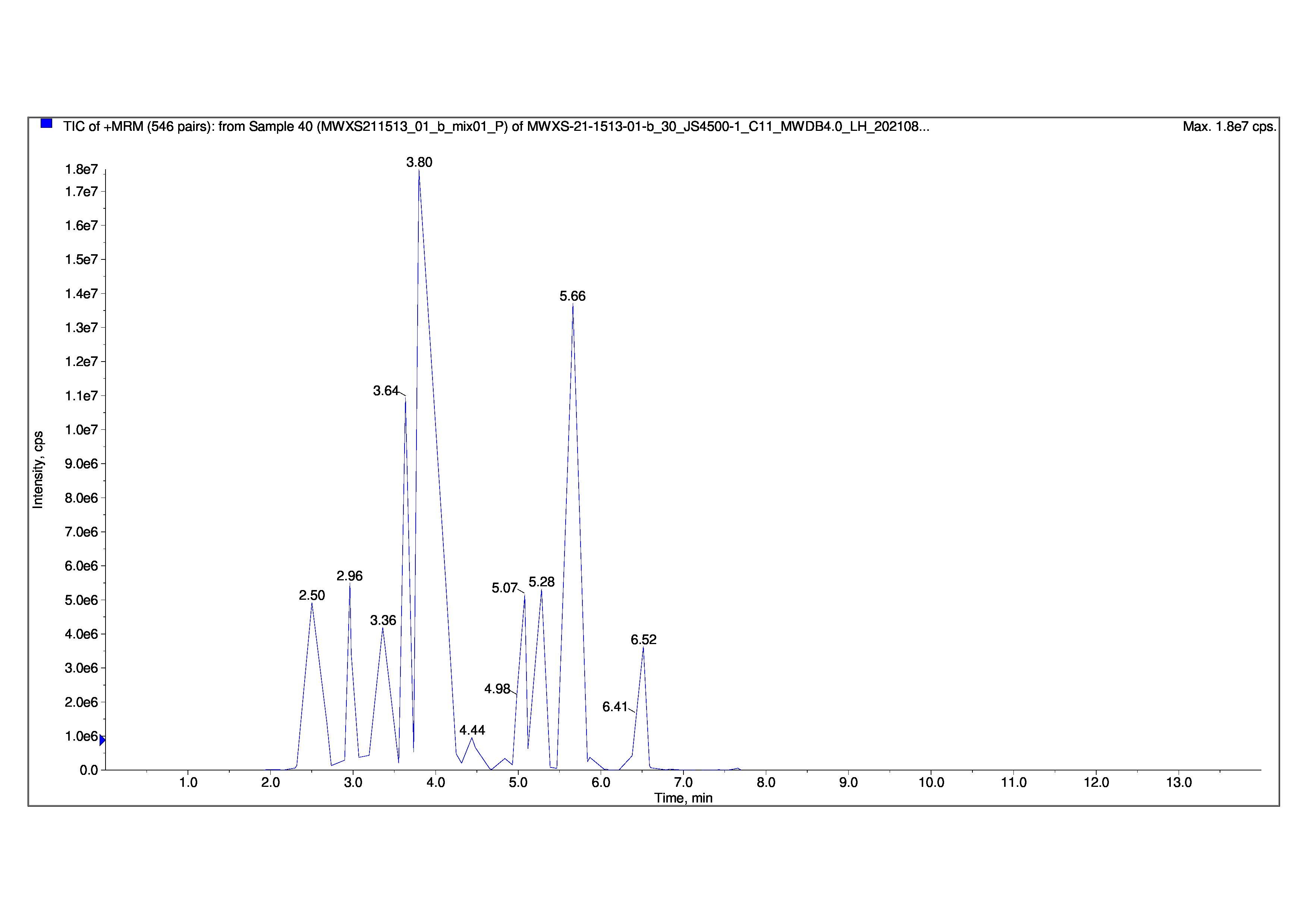


Figure. S1 Analysis of total ion flow diagram of mixed samples by mass spectrometry

Note: Abscissa is the retention time of metabolite detection (Retention time, Rt), ordinate is the ion current intensity of ion detection (cps, count per second).


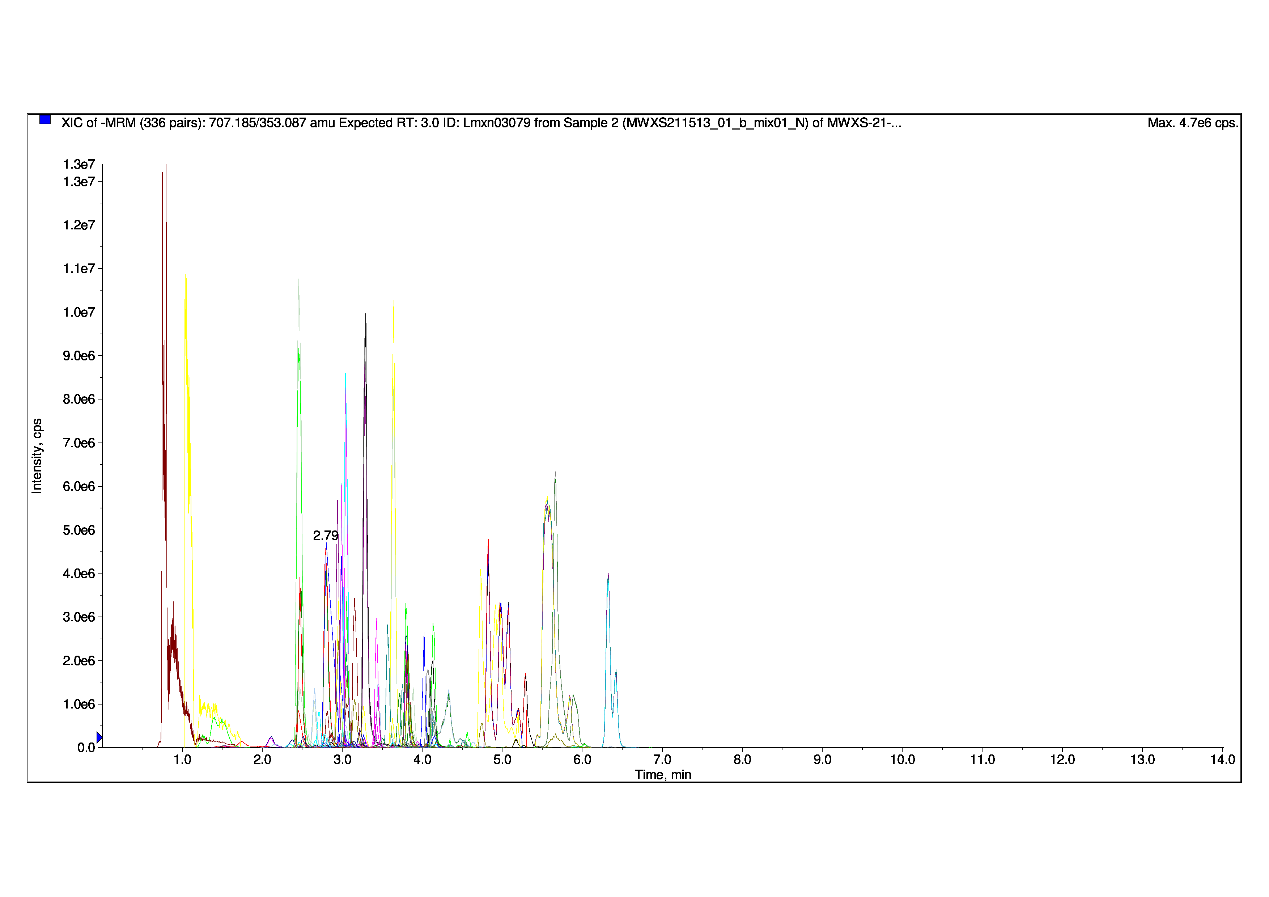

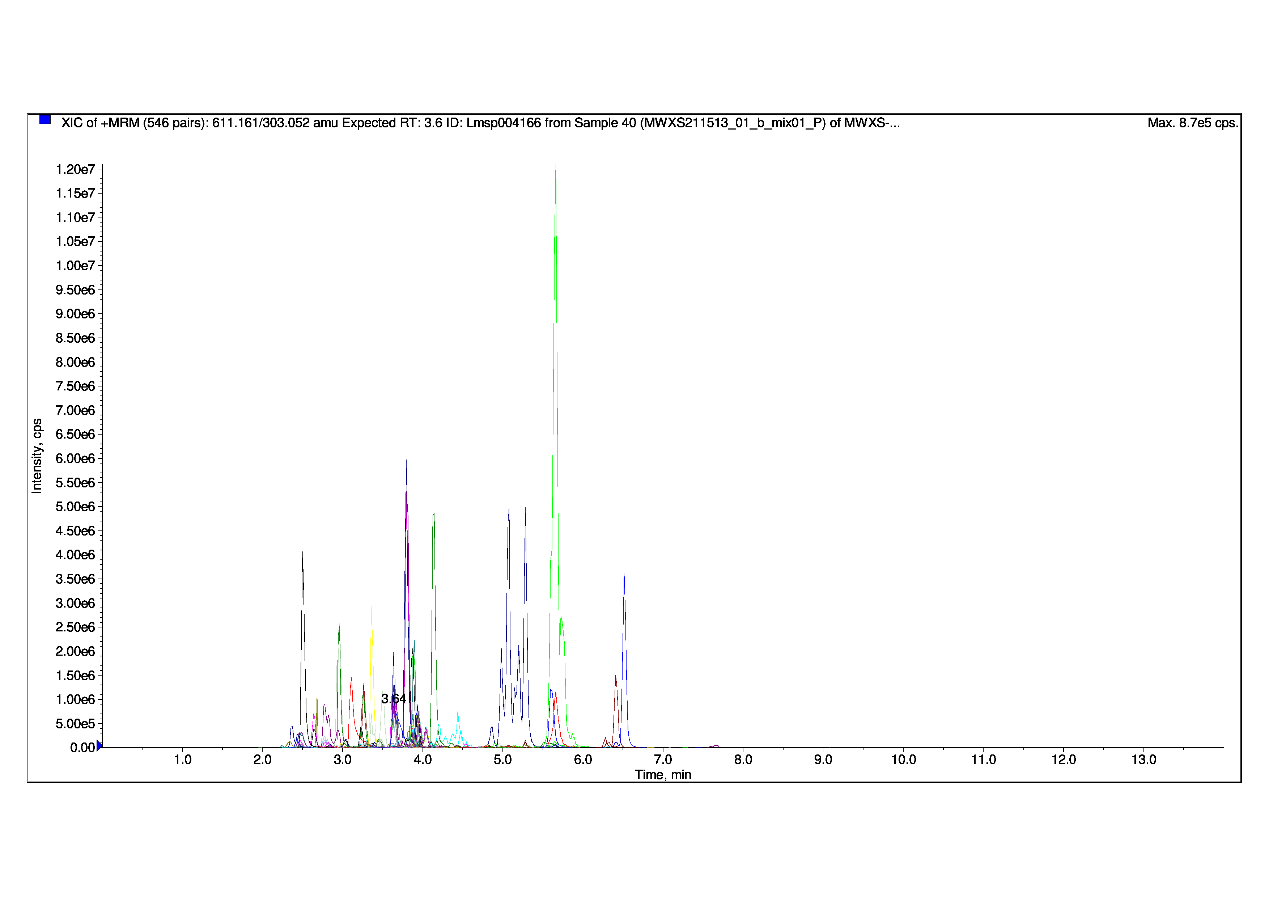


Figure. S2 Multi-peak map of MRM metabolite detection

Note: Abscissa is the retention time of metabolite detection (Retentiontime,Rt), ordinate is the ion current intensity of ion detection (unit of intensity is cps,countpersecond). Each different color of the mass spectrum peak represents a metabolite detected.


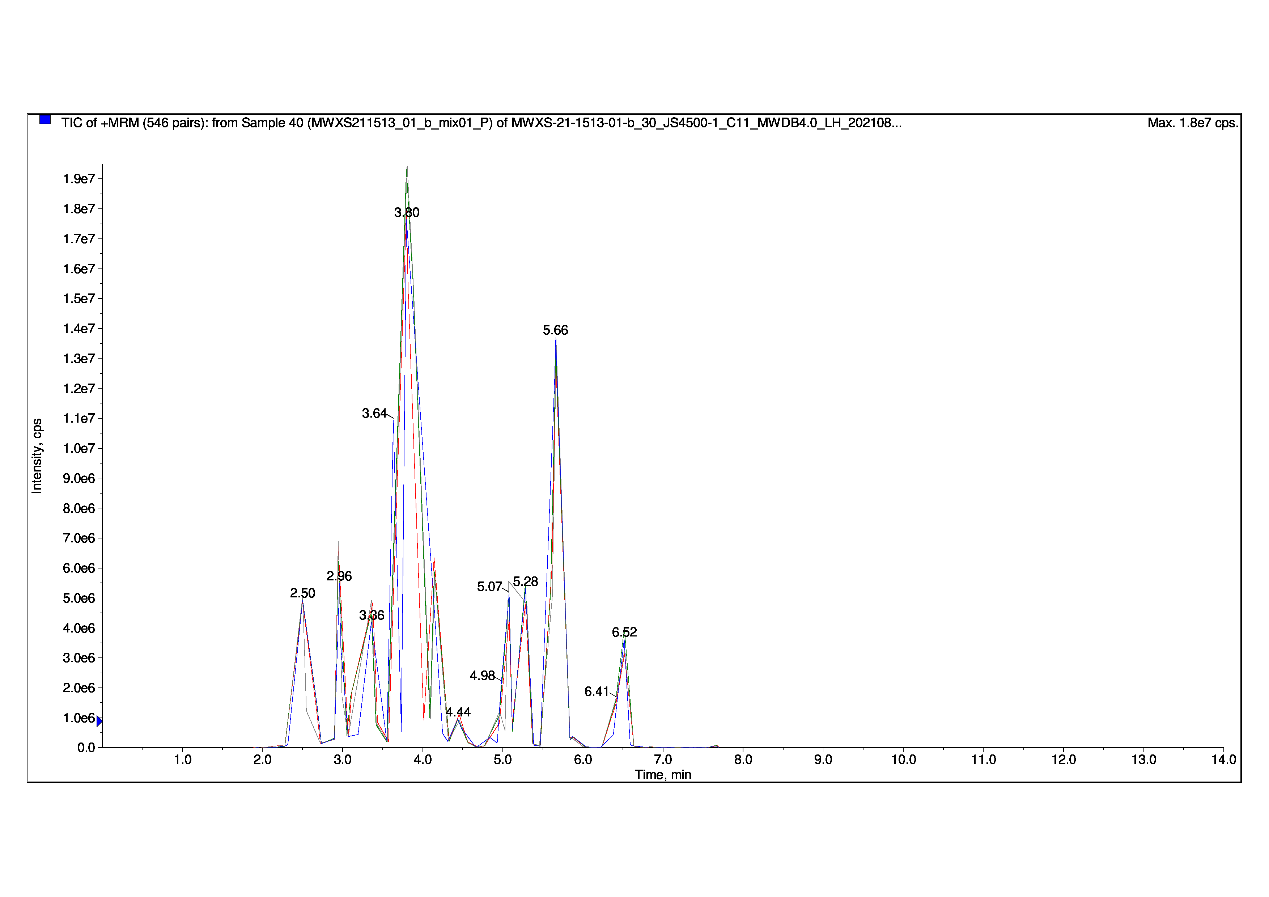

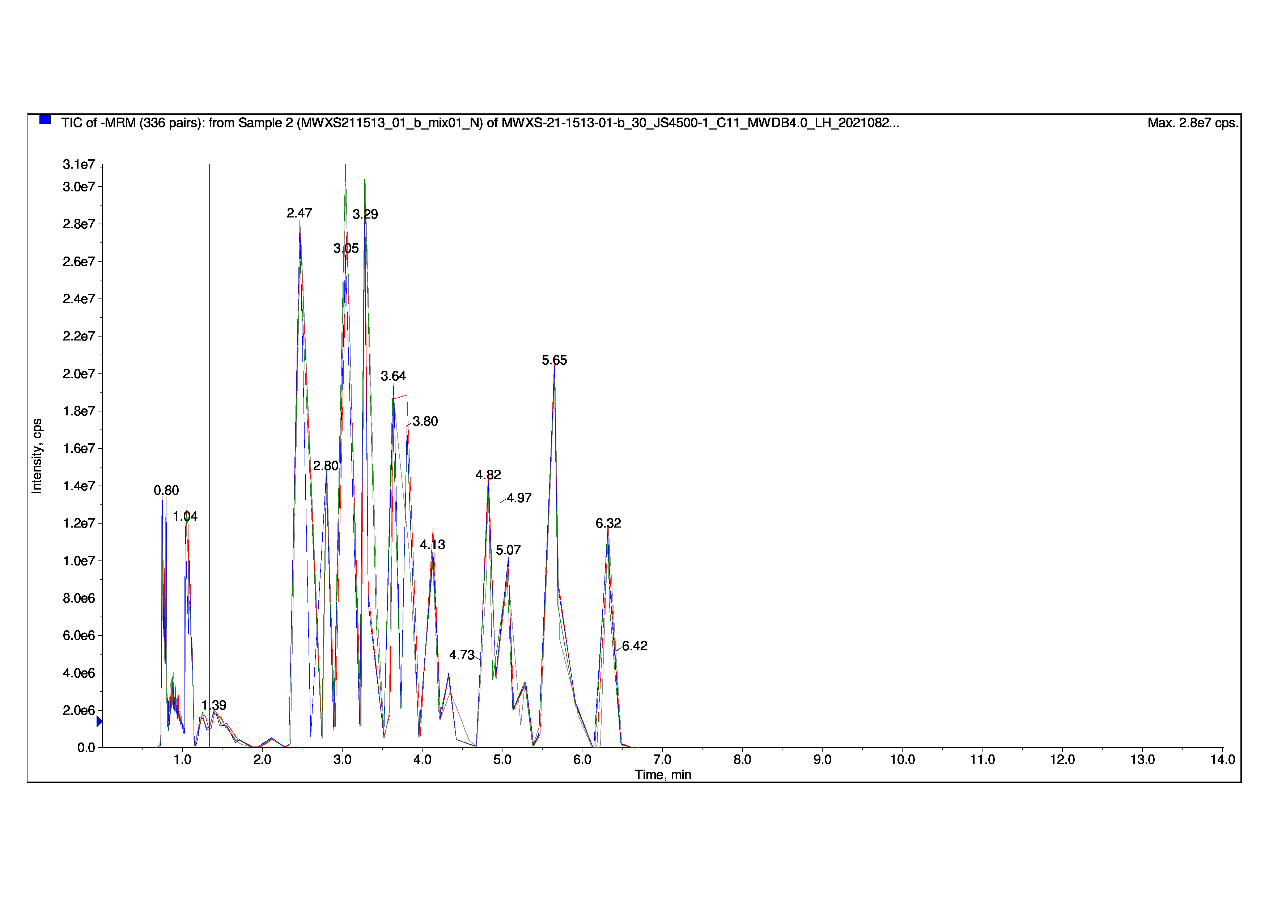


Figure. S3 TIC overlap map of QC samples detected by mass spectrometry

Note: Abscissa is the retention time of metabolite detection (Retentiontime,Rt), ordinate is the ion current intensity of ion detection (unit of intensity is cps,countpersecond).


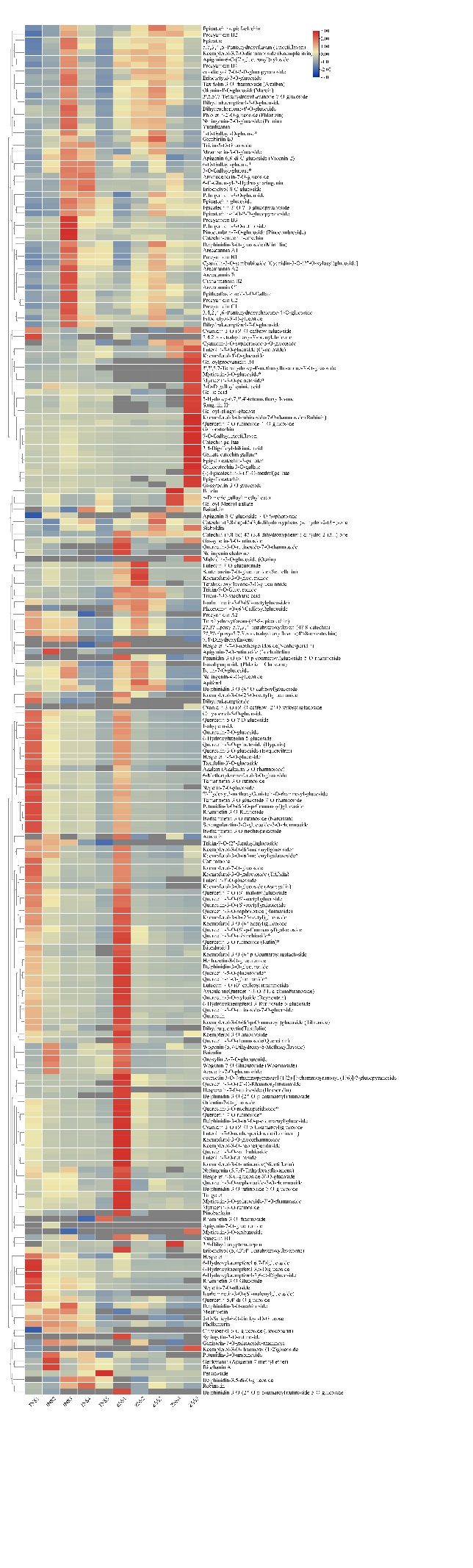


Figure. S4 Heat map of metabolite clustering of two apricot species


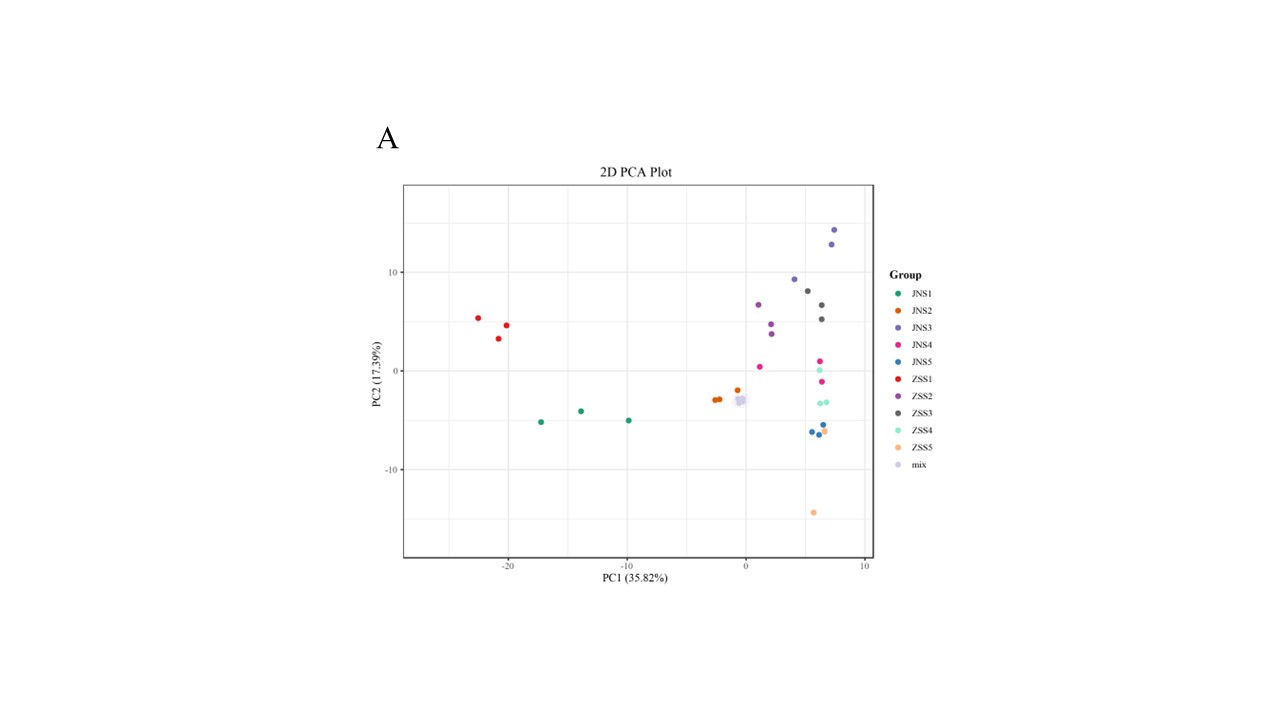

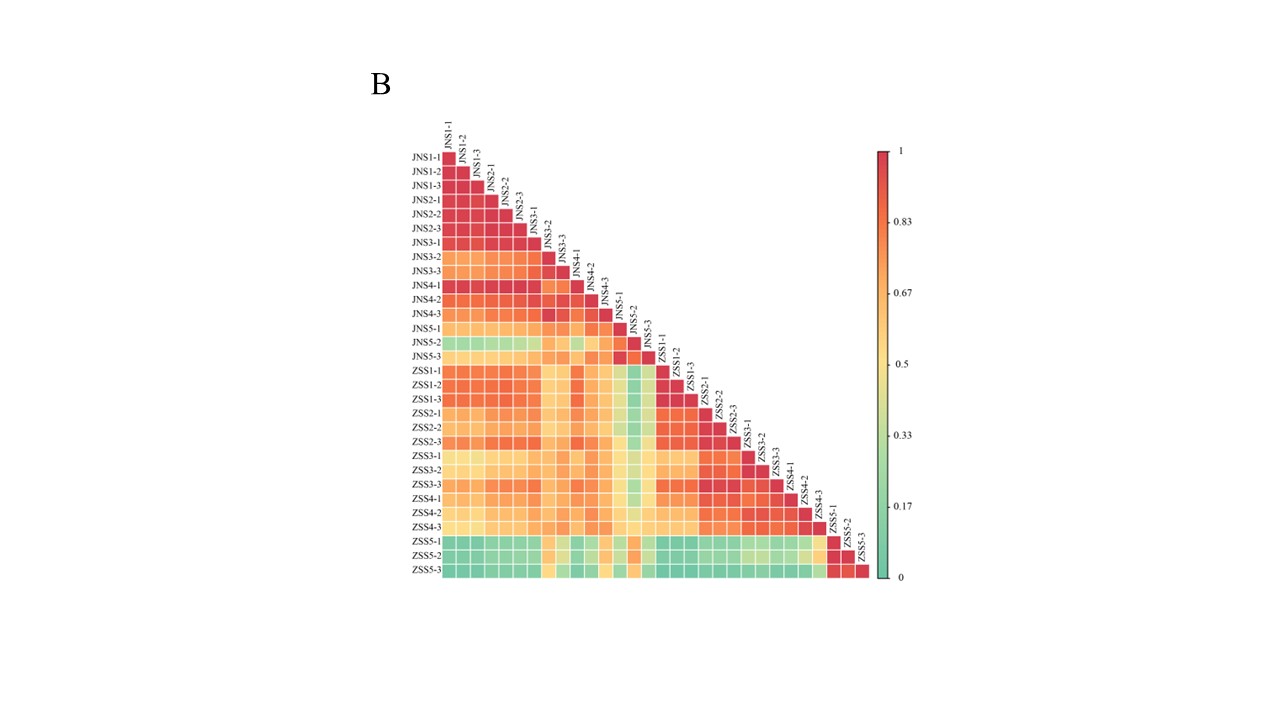


A

B


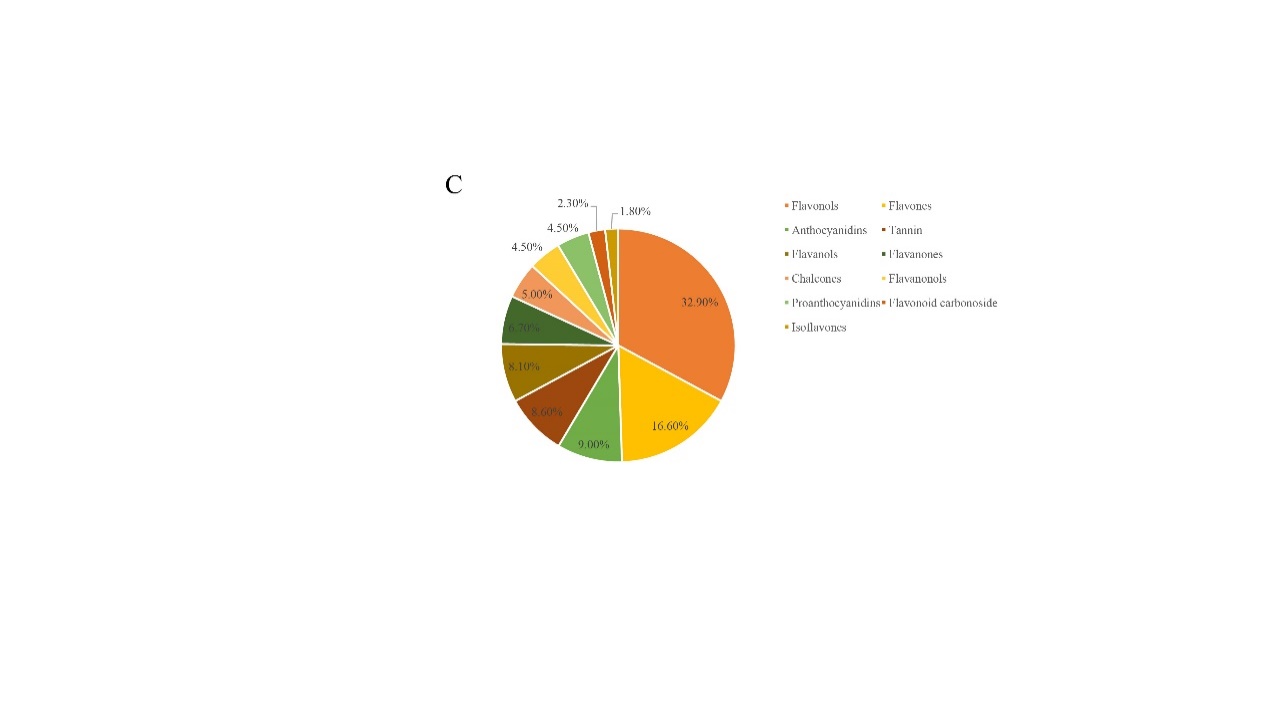


Figure S5 Analysis of metabolites in two apricot fruits at different fertility stages

Note: Graph A, plot of principal component analysis; Graph B, plot of correlation analysis; Graph C, plot of metabolite species analysis. Three replicates were set for each sample.


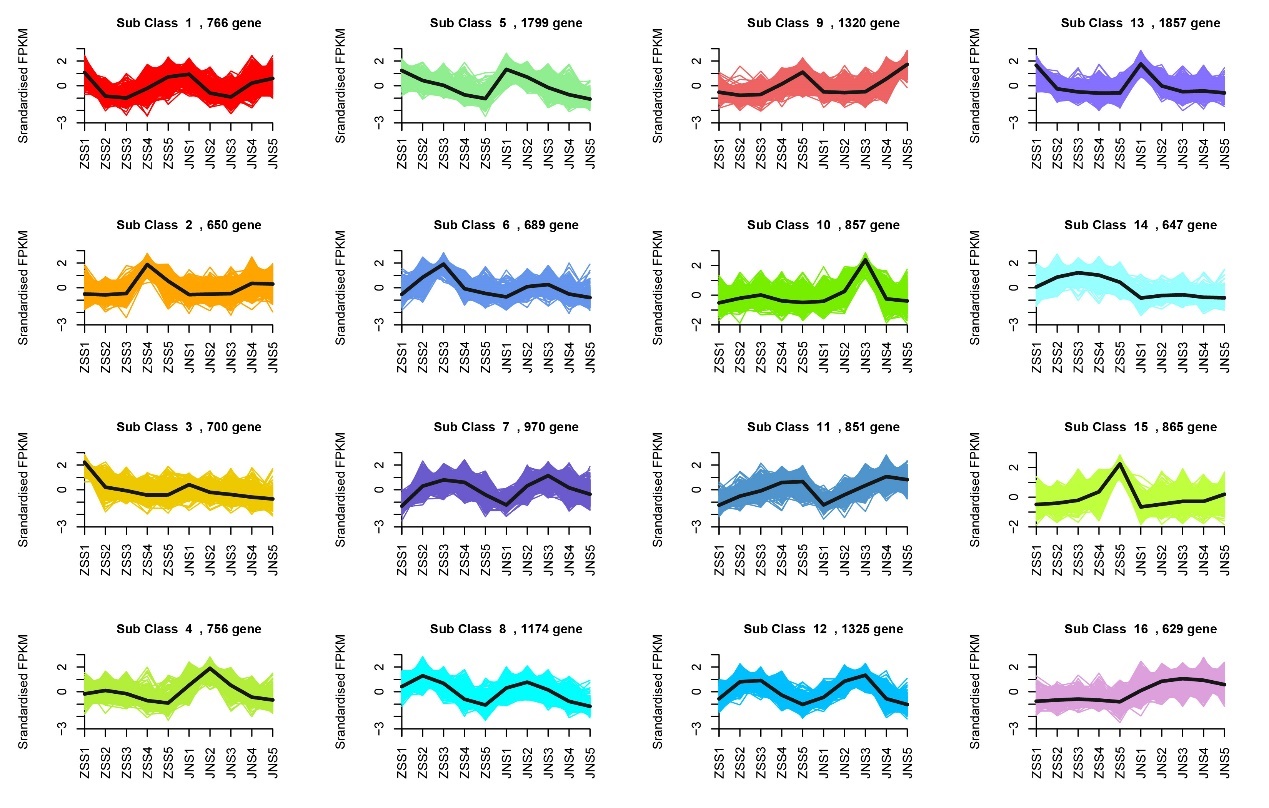


Figure S6 Gene expression difference clustering plot


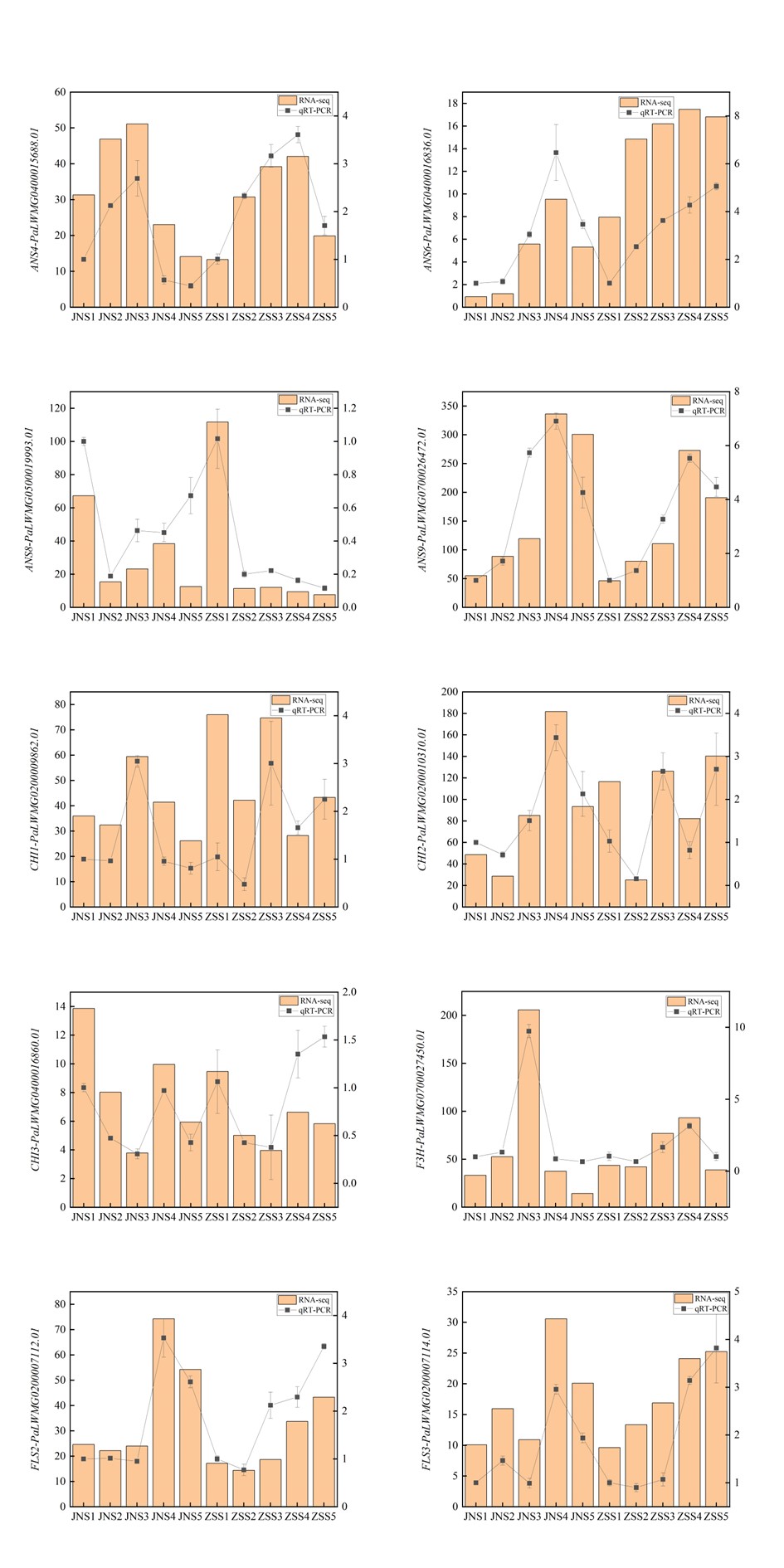


Figure S7 Correlation analysis between transcriptome data and real-time fluorescent quantitative PCR data
